# Crystal structure of a soluble fragment of poliovirus 2C^ATPase^

**DOI:** 10.1371/journal.ppat.1007304

**Published:** 2018-09-19

**Authors:** Hongxin Guan, Juan Tian, Chu Zhang, Bo Qin, Sheng Cui

**Affiliations:** MOH key Laboratory of Systems Biology of Pathogens, Institute of Pathogen Biology, Chinese Academy of Medical Sciences & Peking Union Medical College, Beijing, China; CSIC/IBMB, SPAIN

## Abstract

Poliovirus (PV) 2C^ATPase^ is the most studied 2C protein in the *Picornaviridae* family. It is involved in RNA replication, encapsidation and uncoating and many inhibitors have been found that target PV 2C^ATPase^. Despite numerous investigations to characterize its functions, a high-resolution structure of PV 2C has not yet been determined. We report here the crystal structure of a soluble fragment of PV 2C^ATPase^ to 2.55Å, containing an ATPase domain, a zinc finger and a C-terminal helical domain but missing the N-terminal domain. The ATPase domain shares the common structural features with EV71 2C and other Superfamily 3 helicases. The C-terminal cysteine-rich motif folds into a CCCC type zinc finger in which four cysteine ligands and several auxiliary residues assist in zinc binding. By comparing with the known zinc finger fold groups, we found the zinc finger of 2C proteins belong to a new fold group, which we denote the “Enterovirus 2C-like” group. The C-terminus of PV 2C^ATPase^ forms an amphipathic helix that occupies a hydrophobic pocket located on an adjacent PV 2C^ATPase^ in the crystal lattice. The C-terminus mediated PV 2C-2C interaction promotes self-oligomerization, most likely hexamerization, which is fundamental to the ATPase activity of 2C. The zinc finger is the most structurally diverse feature in 2C proteins. Available structural and virological data suggest that the zinc finger of 2C might confer the specificity of interaction with other proteins. We built a hexameric ring model of PV 2C^ATPase^ and visualized the previously identified functional motifs and drug-resistant sites, thus providing a structure framework for antiviral drug development.

## Author summary

Since the launch of the Global Polio Eradication Initiative, the number of poliomyelitis cases has significantly reduced but obstacles to disease eradication remain. In the endgame phase, anti-poliovirus drugs will be critical in controlling transmission of vaccine-derived polioviruses and in treating patients with chronic infection. However, no effective anti-poliovirus drugs are yet available. The 2C^ATPase^ encoded by poliovirus is one of the most important drug targets, and many inhibitors of 2C have been found. We report here the crystal structure of a soluble portion of PV 2C^ATPase^, containing an ATPase domain, a zinc finger and a C-terminal helical domain. Our findings not only revealed common and individual structural features in the picornaviral 2C protein family, but also allowed us to visualize a large collection of functional motifs and drug-resistant sites identified from the decades-long investigations of poliovirus 2C^ATPase^. Our findings are invaluable for understanding the function of picornavirus 2C proteins and the development of antiviral drugs.

## Introduction

Poliovirus (PV) is the pathogen of poliomyelitis. PV infection can directly result in damage of motor neurons and cause neural lesions [1]. Since the launch of Global Polio Eradication Initiative (http://polioeradication.org/) by the World Health Assembly, the number of poliomyelitis cases have been significantly reduced. The incidence of paralytic polio in 1988 was 1,000 children per day, and this number decreased to 400 per day in 2013[2]. Only three endemic countries remain today. Nevertheless, obstacles to global polio eradication remain. To overcome the last hurdles in the endgame phase, effective anti-PV drugs are critical in controlling transmission of vaccine-derived polioviruses (VDPVs) and in treating patients with chronic infection or personnel casually exposed to PV [3]. Further, to minimize poliomyelitis risk in the “post-polio” era, the National Research Council of the Unite States concluded that the development of antiviral drugs would be important, and possibly essential [4].

PV belongs to Enterovirus C species in the *Enterovirus* genus, *Picornaviridae* family [5]. It has an icosahedral non-enveloped capsid containing a single-stranded +RNA genome of ~ 7,500 nucleotides. PV has three serotypes, PV1 PV2 and PV3, whose capsids exhibit distinct antigenicity [6], but the replicative enzymes of PV are highly conserved not only among different serotypes, but also in other virus species. Therefore, the virally encoded replicative enzymes are considered as good targets for broad-spectrum antiviral drugs [7].

Among EVs replicative enzymes, 2C protein is arguably the most attractive target for direct-acting antivirals (DAA) development [8]. At least ten inhibitors have been found targeting 2C, including guanidine hydrochloride (GuHCl) [9,10], HBB [11], TBZE-029 [12], MRL-1237 [13,14], pirlindole, dibucaine, zuclopenthixol [15], hydantoin [16], fluoxetine [17,18] and brefeldin A [19]. Genotype analyses of drug-resistant virus clones had revealed a large set of residues of 2C that are likely to be involved in binding. Structural characterization of 2C proteins is therefore essential to visualize these potential drug binding sites and to assist antiviral drug development.

2C protein of enteroviruses has typically ~330 residues. It is composed of a N-terminal membrane binding domain, a central ATPase domain, a cysteine-rich domain and a C-terminal helical domain [20]. We recently determined the crystal structure of a soluble portion of 2C helicase from human enterovirus 71 (EV71) [21], the first high-resolution 2C structure in *Picornaviridae*. The ATPase domain of EV71 2C exhibits the structural characteristics of superfamily 3 (SF3) helicases. EV71 2C has an unusual zinc finger with three cysteine ligands. The C-terminus of EV71 2C forms an amphipathic helix that mediates self-oligomerization through a specific interaction between 2C-2C. The hexameric ring model of EV71 2C revealed that the central pore is negatively charged and a putative RNA binding motif is located on the rim of the ring, which suggests the mode of RNA binding might be the winding around the hexameric ring.

Poliovirus (PV) 2C^ATPase^ has been intensively studied for decades. Numerous genetic and biochemical studies have shown that PV 2C^ATPase^ is implicated in series of events in virus life-cycle, from uncoating [22], cellular membrane rearrangement and assembly of membranous replication complexes [23–27], viral RNA synthesis [28–30] to morphogenesis [31–34]. PV 2C^ATPase^ has only 329 residues but is very rich in features. It can be divided into an N-terminal domain, a central ATPase domain and a C-terminal domain. The N-terminal domain harbors a membrane binding motif [35], an amphipathic helix [30], an oligomerization motif [36] and an RNA binding motif [37]. The extreme N-terminal sequence of 2C is able to interact both with virally encoded proteins, 2B, 2BC, 3A, 3AB, 3C, VP3 [20,32,38,39] and cellular protein RTN3 [40]. Structural investigation of PV 2C^ATPase^ has been challenging because the N-terminal domain confers protein insolubility. Our structural characterization of EV71 2C was possible only when the N-terminal domain was removed. We characterized the roles of Walker A + B motifs, Motif C and arginine finger (R-finger) of EV71 2C in ATP hydrolysis and virus production. All of these conserved ATPase motifs are present in PV 2C^ATPase^, and should exert similar functions [41,42]. It was predicted that the C-terminal cysteine rich motif of PV 2C^ATPase^ forms a CCCC type zinc finger [43], but the second potential zinc coordination sites (PCS) of the putative zinc finger is not present in many other enterovirus 2C proteins including EV71 2C. The crystallographic data showed that EV71 2C indeed lacks the PCS2 and it possesses a rare zinc finger with only three cysteine ligands, but it does not seem to affect its zinc coordinating capability [21]. Whether PV 2C^ATPase^ has a distinct zinc finger requires structural investigation. PV 2C^ATPase^ shares 63.7% amino acid sequence identity with EV71 2C helicase, however, no conclusive evidence has shown that PV 2C^ATPase^ has helicase activity to date. To support RNA synthesis, Cho et al found that the 3D polymerase of poliovirus has the activity of unwinding long stretch of RNA duplex, and the nascent RNA strand can be displaced from the template during chain elongation reaction[44]. Thus, the unwindase activity of PV 3D satisfies the necessity of using a helicase during PV replication.

Although many inhibitors have been found targeting PV 2C^ATPase^ over decades, the high-resolution structure of PV 2C^ATPase^ was still missing to date, which hindered the understanding of the mechanisms of inhibitor’s efficacy and drug-resistance and the development of anti-PV drugs. GuHCl for example inhibits ATPase activity of PV 2C^ATPase^ *in vitro* and *in vivo* [42]. Mutations that confer resistance to GuHCl were mostly mapped in segments outside the catalytically important ATPase motifs [9,10], whose precise functions were unknown.

We report here the crystal structure of a soluble fragment of PV 2C^ATPase^ containing the complete ATPase and the C-terminal domains to a resolution limit of 2.55Å. We found that the C-terminal helix mediates self-oligomerization of PV 2C^ATPase^ via a specific interaction between 2C-2C, which is essential to ATPase activity. Comparing the structures of PV 2C and EV71 2C revealed both common and distinct structural characteristics. We built a hexameric ring model of PV 2C^ATPase^ to visualize regions important to PV 2C^ATPase^ function and drug resistance.

## Results

To gain atomic insight into poliovirus 2C^ATPase^, we carried out a crystallographic study. We first expressed a N-terminal maltose-binding protein (MBP) tagged full-length 2C^ATPase^ from human poliovirus 1 (strain Mahoney, GenBank: KU866422.1); however, the MBP tagged protein did not yield crystals. Removal of MBP tag by proteinase cleavage only led to precipitation of PV 2C^ATPase^. Therefore, we employed a similar strategy as used in expressing the soluble fragment of EV71 2C [21]. Firstly, we removed the N-terminal 115 residues of PV 2C^ATPase^, (PV-2C-ΔN). This fragment lacks the N-terminal membrane binding domain but retains a complete ATPase domain, a zinc binding site and a C-terminal helical domain. PV-2C-ΔN was soluble, but still failed to crystallize. Considering that surface-entropy reduction may favor crystallization [45], we predicted a set of surface residues charges based on EV71 2C structure [21]. We then introduced alanine substitutions systematically to these residues with an aim of minimizing surface charging. We finally obtained crystals of PV-2C-ΔN bearing mutations E207A, K209A and R149A (PV-2C-ΔN-3Mut). None of these mutations is located within conserved ATPase motifs, zinc binding site or the C-terminal helical domain.

**Fig 1:**
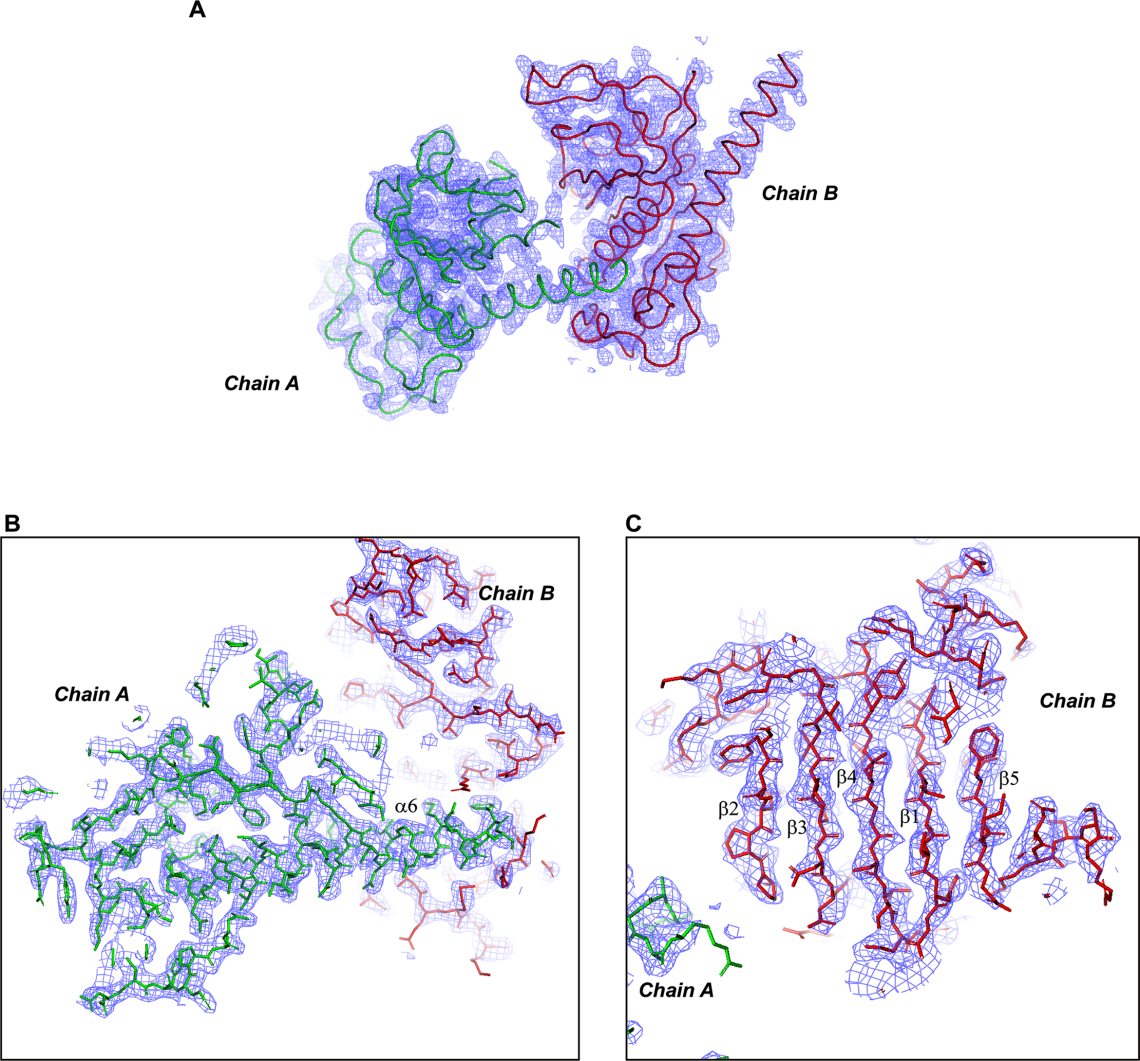
A simulated-annealing composite omit map of PV-2C-ΔN-3Mut. A composite omit map of the 2.55Å data of PV-2C-ΔN-3Mut crystal structure was calculated using the software phenix.composite_omit_map. The omit map was generated by running the software in the simulated-annealing mode. The annealing method used was cartesian with an annealing temperature of 5,000K. This is a 2Fo-Fc map contoured at 1.2 σ. (A) The simulated-annealing composite omit map for chain A and B is shown with the blue mesh; the ribbon model of chain A (green) and B (red) are superimposed with the map. (B) A magnified view of the core and the C-terminal long helix of chain A; the stick model of the structure is superimposed. (C) A magnified view of the core of chain B, illustrating the five-stranded parallel β-sheet plane of the ATPase domain of PV 2C; the stick model of the structure is superimposed.

We grew PV-2C-ΔN-3Mut crystals in a hanging drop vapor diffusion system at 20°C. The crystals diffracted the X ray to a 2.55Å. It belonged to the space group of P2_1_ and contained 6 copies of 2C per asymmetric unit (ASU). We solved the crystal structure of PV-2C-ΔN-3Mut by molecular replacement using EV71 2C structure (PDB code: 5GQ1, B chain) as the search model. Several regions of the initial atomic model were built manually, especially at the zinc binding site that accounted for the largest structural discrepancy. In the finally refined model, we were able to located most of residues in chains A, B, C, D and E. But chain H was largely disordered, only a fraction of residues was visible in the electron density. Chain A and B were the most intact 2C copies and they were associated with relatively low temperature factors, 67.82 Å^2^ and 72.44 Å^2^, respectively. By contrast, the temperature factors of the other chains C, D, E and H are much higher; the values are 108.38 Å^2^, 76.55 Å^2^, 105.38 Å^2^ and 170.56 Å^2^, respectively. Therefore, only chain A and B were used for further structural analysis. Because the average temperature factor of this structure is high, we generated a simulated-annealing 2Fo-Fc composite omit map to validate its quality. As illustrated in Fig 1, the simulated-annealing composite omit map has a good fit with the final model PV-2C-ΔN-3Mut, which supported that the model was correctly built. We performed a structural comparison of the chains within ASU (S1 Fig). The r.m.s.d. values among different chains ranges from 0.4Å to 1.3Å. The largest structural deviation is contributed by a small loop region 180-184aa of B chain between β3 and α2, which is apparently resulted from the crystal packing artifacts. The C-terminal helical domain of PV 2C exhibits only slightly different orientation, but this is not comparable with the dramatical conformational changes observed at the C-terminal helix in EV71 2C structure[21] (PDB entries: 5GQ1 and 5GRB). The statistics of data collection, structure refinement and structure validation are summarized in Table 1.

**Table 1:** Data collection and refinement statistics.

	PV-2C-ΔN-3Mut, residues 116–329 (PDB ID: 5Z3Q)
**Data collection**	
Space group	P 2_1_
Cell dimensions	
a, b, c (Å)	52.70, 84.07, 172.56
α, β, γ (°)	90.00, 94.21, 90.00
X ray source	SSRF BEAMLINE BL18U1
Wavelength (Å)	0.98
Data range (Å)	43.81–2.55
Reflections unique	95988 ^a^
*R*_sym_ ^b^ (last shell)	0.119 (0.949)
*I* / σ*I*	7.56(1.10)
Completeness (%) (last shell)	99.1(96.4)
Redundancy (last shell)	3.42(3.22)
**Refinement**	
Resolution range (Å)	40.62–2.55
Reflections, cut-off, % reflections in cross validation	95842, F>1.34, 5.13
*R*_work_ ^c^/ *R*_free_ ^d^ (last shell)	0.2020/ 0.2650 (0.3059/0.3463)
**Atoms**	
Non-hydrogen protein atoms	8590
Protein	8351
Ligands (ZN or PO_4_)	21
Solvent	218
*B*-factors average (Å^2^)	92.58
Protein (Å^2^)	92.75
Ligands (Å^2^)	89.94
Solvent (Å^2^)	79.73
**r.m.s.d**	
Bond lengths (Å)	0.018
Bond angles (°)	1.471
**Validation**	
MolProbity score	2.52 76^th^ percentile ^e^ (N = 6632, 2.545Å ± 0.25Å)
Clashscore, all atoms	18.63 77^th^ percentile ^e^ (N = 259, 2.545Å ± 0.25Å)
% residues in favored regions, allowed regions, outliers in Ramachandran plot	94.34, 5.27, 0.38

^a^ Friedel pairs are treated as different reflections

^b^ *R*_sym_ = ∑_hkl_∑_j_ |Ihkl,j—I_hkl_|/∑_hkl_∑_j_I_hkl,j_, where I_hkl_ is the average of symmetry-related observations of a unique reflection

^c^ *R*_work_ = ∑_hkl_ ||*F*_obs_(hkl)|-|*F*_calc_(hkl)||/∑_hkl_|*F*_obs_(hkl)|.

^d^ *R*_free_ = the cross-validation *R* factor for 5% of reflections against which the model was not refined.

^e^ 100^th^ percentile is the best among structures of comparable resolution; 0^th^ percentile is the worst. For clashscore the comparative set of structures was selected in 2004, for MolProbity score in 2006.

**Fig 2:**
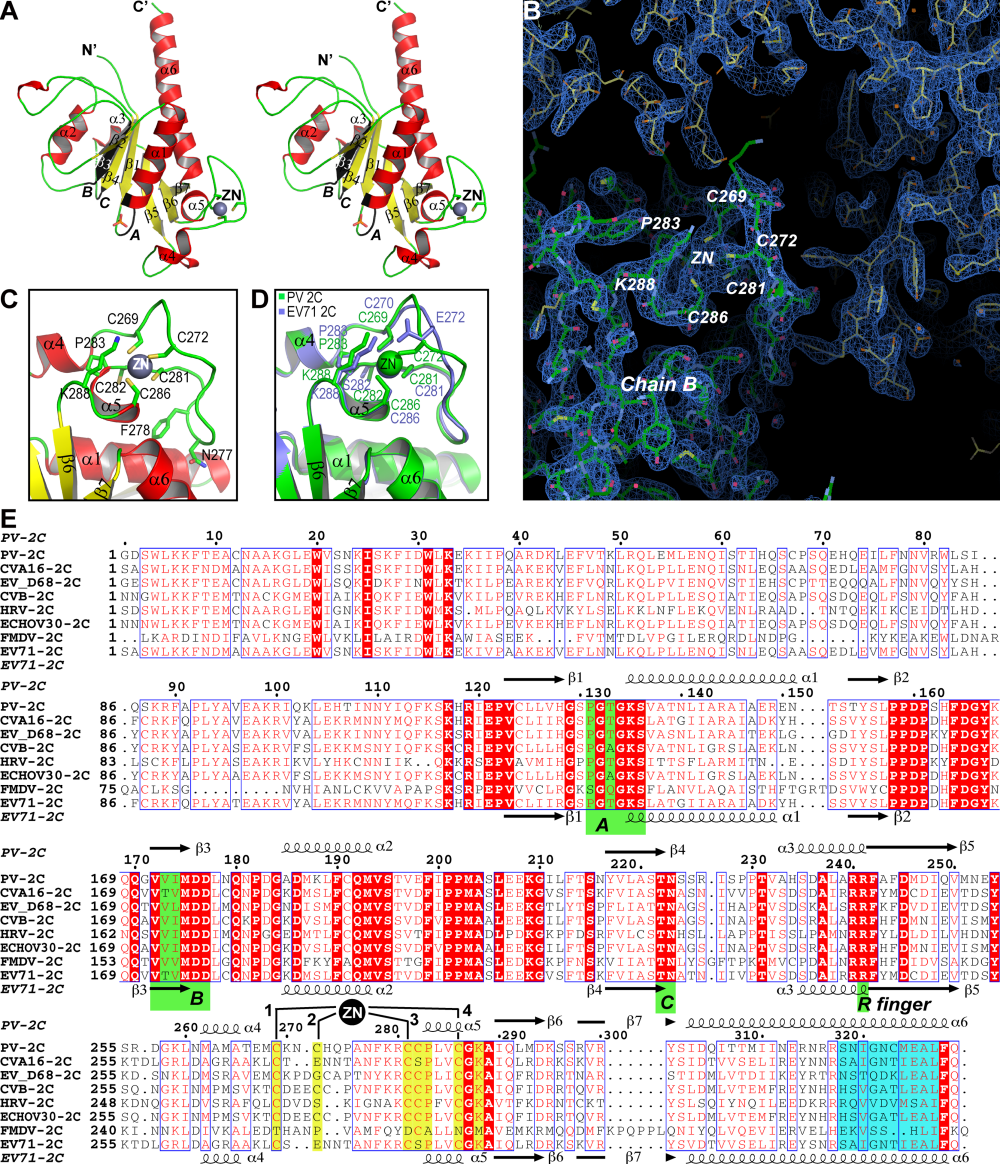
Overall structural features and the zinc finger of PV-2C-ΔN-3Mut. (A) Ribbon model of PV-2C-ΔN-3Mut (wall-eye stereo image). The model is colored by secondary structure elements, α-helices red, β-strand yellow and loops green. Secondary structure elements are labeled. The conserved ATPase motifs, Walker A, Walker B and Motif C are highlighted in black and labeled with *A*, *B* and *C*, respectively. The ligands are: a zinc atom shown as a gray sphere, and a phosphate group shown with stick model in orange. (B) A view of PV 2C zinc binding site of chain B with the final 2Fo-Fc map (blue mesh, contour 1.2 s). The final atomic model of PV-2C-ΔN-3Mut is shown with the stick model. Residues important to zinc coordination are indicated. (C) A magnified view of the zinc finger of PV 2C with the same color scheme as in panel A. Residues involved in zinc binding and stabilizing the folding of the zinc finger are shown with stick model and labeled. (D) Structure alignment of zinc fingers from PV 2C (green) and EV71 2C (blue). Residues involved in zinc binding are shown with stick model and labeled. (E) Structure-based multiple sequence alignment of various picornavirus 2C helicases. Residues highlighted with red background are invariant, residues in red color are conserved. While the secondary structure elements of PV 2C are aligned on top of the sequences, the secondary structure elements of EV71 2C are aligned at the bottom. The conserved ATPase motifs are highlighted with green background with labels. The zinc atom is illustrated with a black disc and four zinc binding sites are marked with 1, 2, 3 and 4, respectively. Residues involved in zinc binding are highlighted with yellow background. Residues forming the pocket binding domain (PBD) are highlighted with blue background.

The overall fold of PV-2C-ΔN-3Mut is similar to EV71 2C. Structural comparison between PV-2C-ΔN-3Mut and EV71 2C (PDB code: 5GRB, chain C) gave a Dali Z-score of 30.9 with 197 aligned Cα atoms and the r.m.s.d. value was 1.3Å. PV-2C-ΔN-3Mut is comprised of an ATPase domain with canonical α/β Rossmann fold, a CCCC type zinc finger followed and a long helical C-terminus (Fig 2A). The ATPase domain contains a five-stranded parallel β-sheet (β1-β5) surrounded by three α-helices (α1-α3). We located three conserved ATPase motifs. Walker A motif is located on the loop connecting β1 and α1, forming the phosphate binding loop (P-loop). A phosphate group was found occupying the P-loop. The Walker B motif is located on the loop between β3 and α2. The SF3 helicase specific motif C is located on the loop between β4 and α3 (Fig 2A and 2E).

The cysteine-rich motif of PV 2C^ATPase^ (residue 267–289), located between α4 and β6, connects the ATPase domain and the C-terminal helical domain. The cysteine-rich motif folds into a CCCC type zinc finger (Fig 2C). The zinc ion is coordinated by four cysteine ligands, C269, C272, C281 and C286. Three of these cysteine residues are from the long loop between α4 and α5, and one is from the C-terminus of α5. The distances from each Sγ atom to the zinc varies from 2.2Å to 2.4Å, which are typical distances between sulfur and zinc as the median distance of Zn-S is 2.28Å [46]. In addition, two conserved residues C282 and K288 assist the zinc coordination. The carbonyl oxygen of C282 buttress the zinc ion from the bottom (distance, 3.3 Å), whereas the side chain Nζ atom of K288 covers the zinc on top (distance 4.2Å) (Fig 2B and 2C). There is an unusually long loop region between the second and third zinc ligands C272 and C281, on which N277 and F278 dock their side chains into a hydrophobic groove formed between α1 and α6 helices. This hydrophobic interaction between the zinc finger and the ATPase domain may serve to further stabilize the folding of the zinc finger (Fig 2C).

Pfister and colleagues predicted four potential zinc coordination sites (PCS1-4) in the cysteine-rich motif of PV 2C^ATPase^ [43]. We therefore adopted this nomenclature in our structural analysis of zinc finger structure for the convenience of comparing with the results reported previously. We found that while the conformation of C269 (PCS1), C281 (PCS3) and C286 (PCS4) are well conserved in both structures (Fig 2D), residue C272 acts as PCS2 for zinc binding in PV 2C^ATPase^, but its structural counterpart E272 in EV71 2C is not a zinc ligand. Instead, E272 forms a salt bridge with K288 stabilizing the overall folding of the zinc finger of EV71 2C. The loop harboring PCS2 (residues 270–276) is among the least conserved regions in picornavirus 2C proteins (Fig 2E), which coincides with the pronounced structural difference of this region between PV 2C^ATPase^ and EV71 2C (Fig 2D).

**Fig 3:**
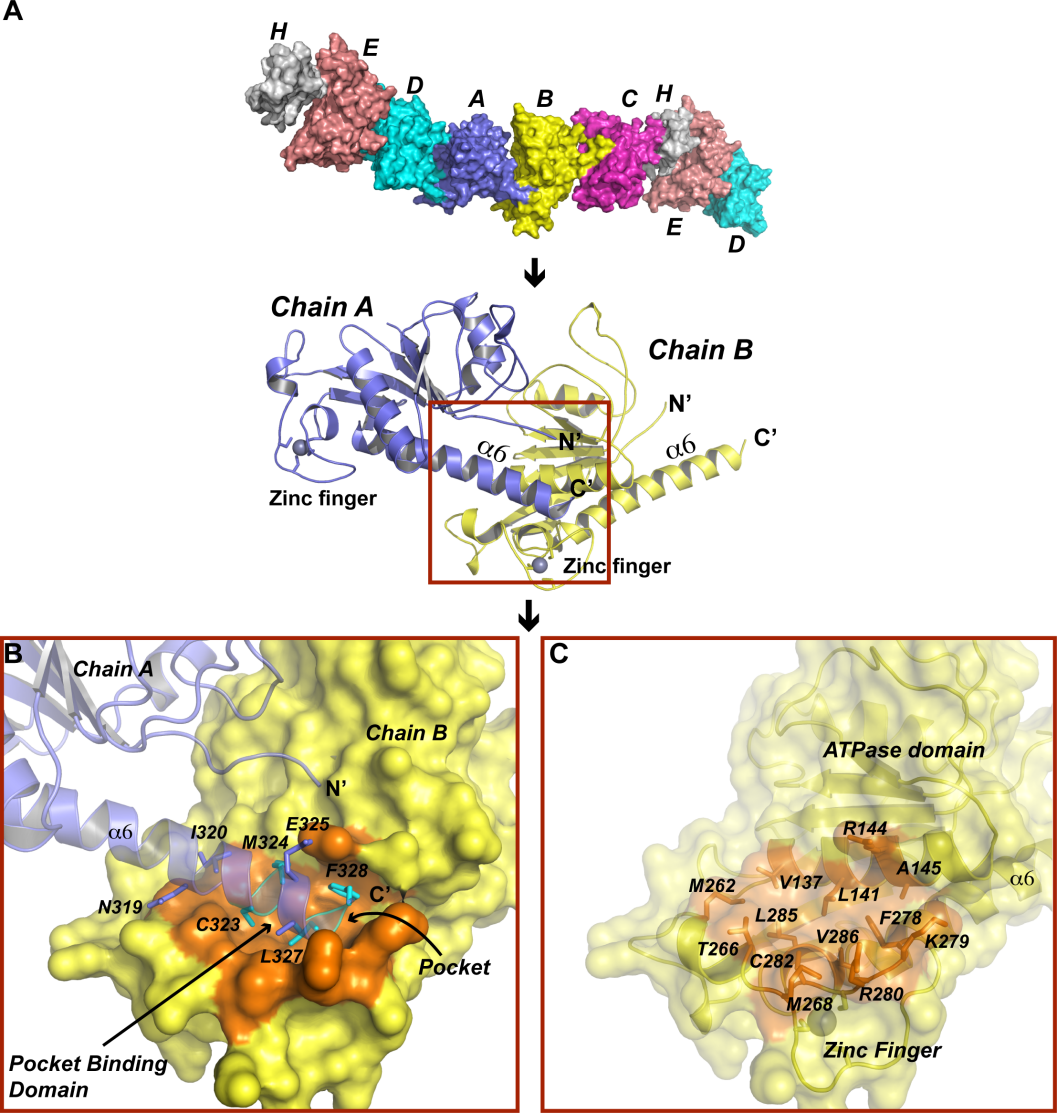
PV-2C-ΔN-3Mut self-oligomerizes in crystals. (A) In the crystal structure of PV-2C-ΔN-3Mut, all 2C copies oligomerize via a specific interaction between the adjacent 2C-2C molecules. Upper part, six chains (A-H) of PV-2C-ΔN-3Mut are shown with molecular surface. They interact with one another, forming continuous polymers in crystal. Lower part, ribbon models of A and B chains. The C-terminus α6 helix of chain A docked inside a pocket on chain B. (B) Magnified view of 2C-2C interaction. The C-terminus α6 helix of PV 2C accommodates a hydrophobic pocket (highlighted in orange) on chain B. Residues forming the pocket binding domain (PBD) are colored in cyan. PBD are buried inside the pocket. Additional interaction between the PBD and the pocket includes a salt bridge between E325 and R144. (C) Same view as in panel B with chain A removed. The molecular surface of chain B is semi-transparent to visualize the structural details of the hydrophobic pocket located between the ATPase domain and zinc finger. Residues constituting the pocket are shown with stick model. The zinc is shown as a gray sphere.

**Fig 4:**
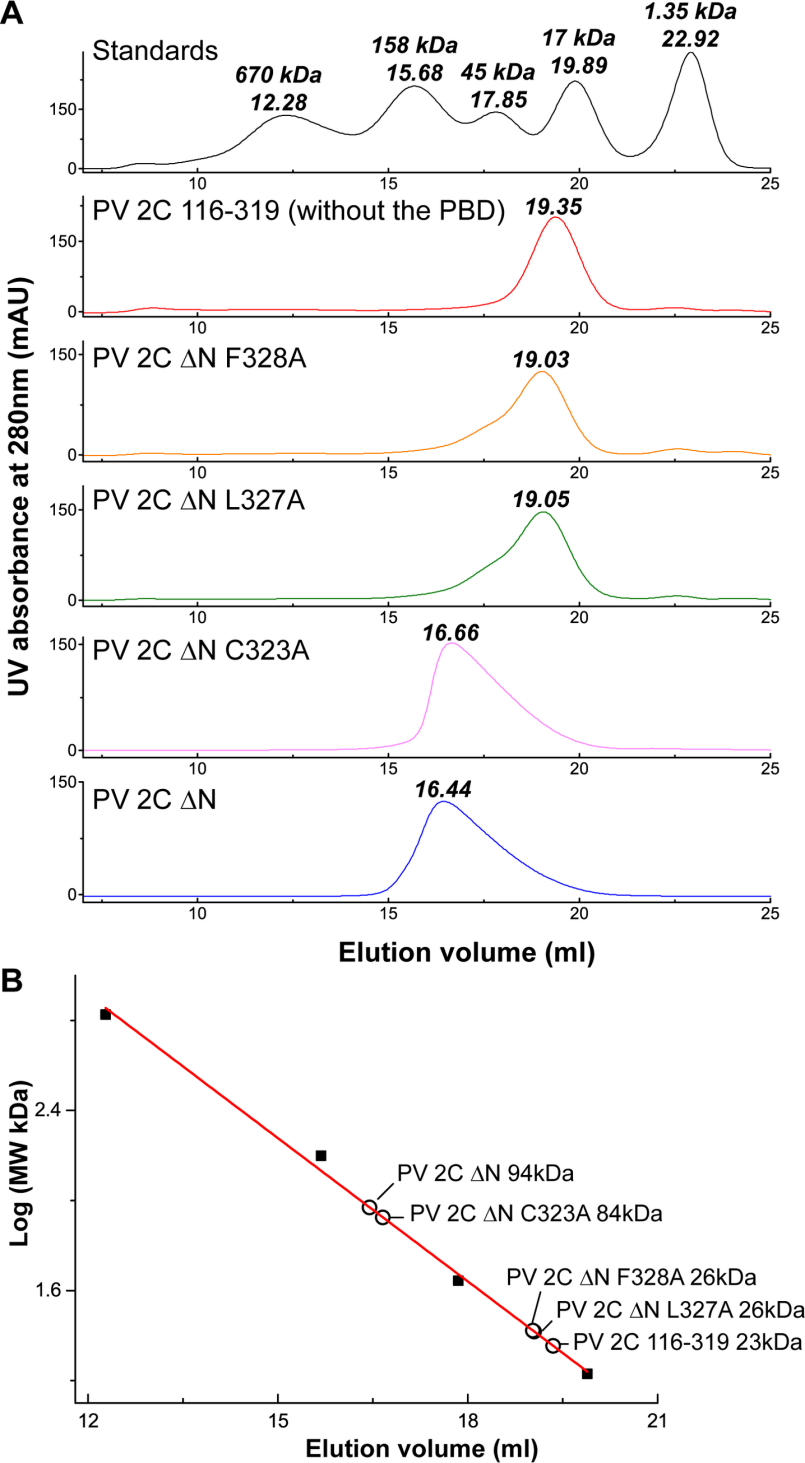
Size exclusion chromatography of PV-2C-ΔN variants. A Superdex200 10/300 GL column was calibrated with a protein standard mix containing thyroglobulin 670kDa, γ-globulin 158kDa, ovalbumin 45 kDa, myoglobin 17 kDa and vitamin B12 1.35 kDa. The molecular mass of a set of PV-2C-ΔN variants were then assessed by size-exclusion chromatography. (A) The profiles of size-exclusion chromatography of the standards proteins (black), PV 2C 116–319 (without the pocket binding domain, red), PV-2C-ΔN F328A (orange), PV-2C-ΔN L327A (green), PV-2C-ΔN C323A (magenta) and PV-2C-ΔN (blue) are aligned. The elution volume of each peak in the profiles are indicated. (B) The Log of the molecular mass (kDa) of the standard proteins is plotted as the function of the elution volume. Four proteins including thyroglobulin, γ-globulin, ovalbumin and myoglobin (filled squares) were used for a linear-fitting to generate a standard curve (red straight line). The molecular mass of different PV-2C-ΔN variants (empty circles) was estimated according to the standard curve. The calculated molecular mass is indicated.

We performed crystal packing analysis of PV-2C-ΔN-3Mut structure and found that all 2C copies in the crystal polymerize via a C-terminus helix mediated 2C-2C interaction (Fig 3A). The mode of PV 2C-2C interaction resembles EV71 2C-2C interaction we characterized previously [21]. Residues C323, M324, L327 and F328 of the C-terminus α6 helix of a PV-2C-ΔN-3Mut monomer formed a pocket-binding domain (denoted: PBD) that docks inside a hydrophobic pocket (denoted: pocket) between the zinc finger and the ATPase helicase domain of an adjacent PV-2C-ΔN-3Mut monomer (Fig 3B). The pocket is formed by 13 residues, most of which are hydrophobic. A salt bridge between E325 from the PBD and R144 from the pocket further stabilizes 2C-2C interaction. To validate PV-2C-ΔN-3Mut oligomerization observed in crystalline state, we investigated self-oligomerization of PV-2C-ΔN using size-exclusion chromatography (Fig 4). The molecular mass of PV-2C-ΔN was observed to be 94kDa, close to the theoretical molecular mass of tetramers 95.2kDa, suggesting PV-2C-ΔN exists as tetrameric in solution. Removing PBD of PV-2C-ΔN (PV 2C 116–319) reduced its molecular mass to 23kDa, nearly matching the theoretical molecular mass for monomers 23.8kDa. Therefore, the tetramerization of PV-2C-ΔN was dependent on the PBD mediated 2C-2C interaction. Our previous results demonstrated that mutation of either L327 or F238 could abolish self-oligomerization of EV71 2C and these two residues are highly conserved among 2C helicases [21] (Fig 2A). We then measured the molecular mass of PV-2C-ΔN bearing mutation L327A and F238A respectively. The molecular mass of these mutants reduced to monomeric size, 26kDa, indicating that both residues play the crucial role in PV 2C^ATPase^ self-oligomerization (Fig 4).

**Fig 5:**
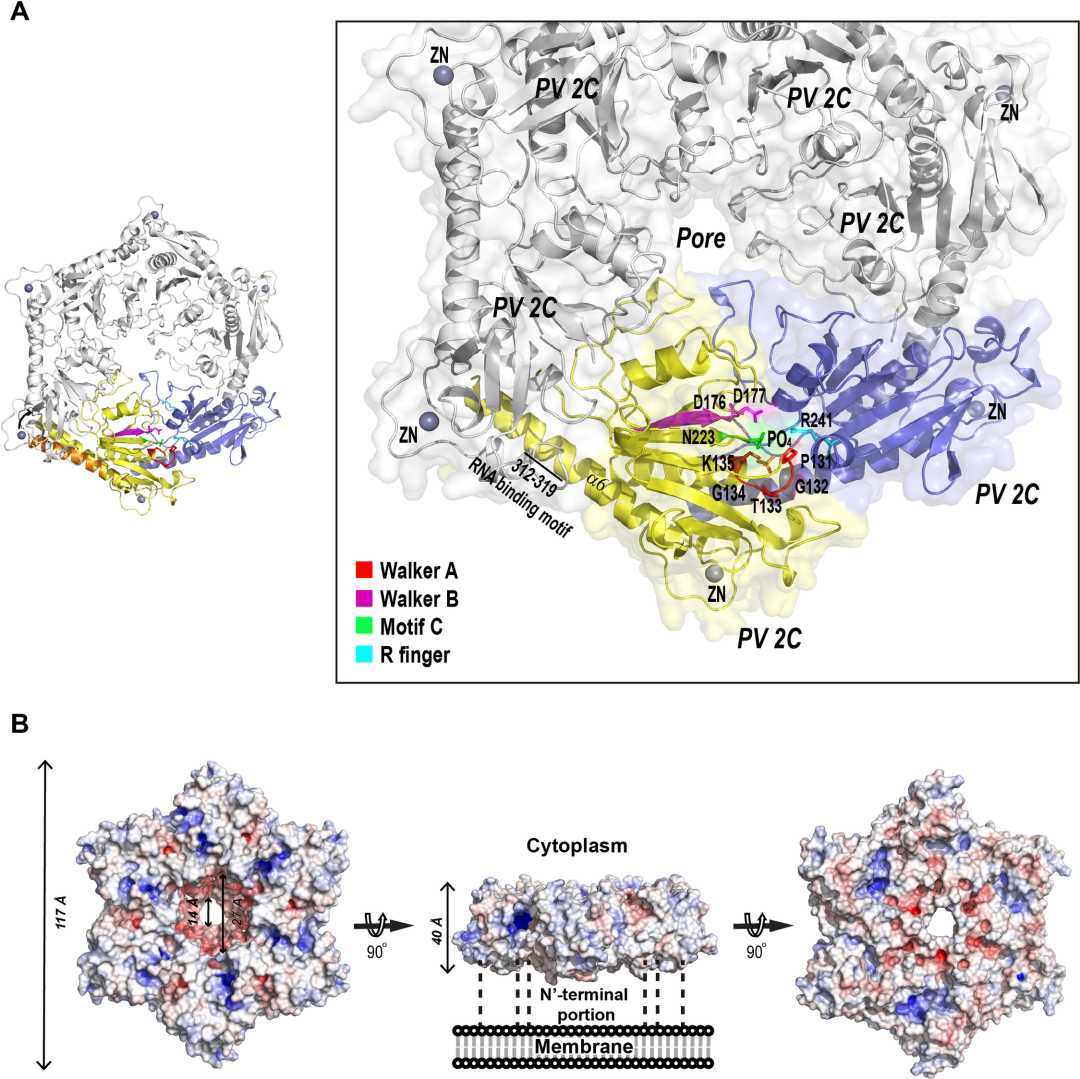
Hexameric model of PV-2C-ΔN. (A) Left, an overview of the hexameric ring model of PV-2C-ΔN. The hexameric ring model was built by superimposing six 2C ΔN monomers onto each subunit of SV40 Large T Antigen (PDB code: 2H1L). The PBD on C-terminus α6 of one 2C is located very close to the hydrophobic pocket on the adjacent 2C; we tilted α6 helix slightly, so that the PBD could fully accommodate the pocket. The α6 colored in orange indicates its conformation before the tilting. Right, a magnified view of the ring on the left with the semitransparent molecular surface. Two adjacent 2C molecules at the bottom of the ring are highlighted in yellow and blue, demonstrating the functional active site conformation between 2C-2C. The conserved ATPase motifs are individually colored, Walker A red, Walker B magenta, Motif C green and R finger cyan. The other 2C monomers are colored in gray. The segment (312–319) important to RNA binding is indicated. (B) Molecular surface of the hexameric ring model of PV-2C-ΔN colored by potential on solvent accessible surface (negative charge, red; positive charge, blue). Left, cytoplasm side of the ring; center, rim of the ring; right, membrane proximal side of the ring. The dimensions of the ring and the funnel shaped central pore are indicated.

Our previous crystallographic study of EV71 2C identified a hinge region on C-terminus α6 helix, which could mediate rotation between 2C-2C, therefore resulting multiple 2C-2C conformations in crystal structure [21]. By contrast, we did not find the similar 2C-2C rotations in the crystal structure of PV-2C-ΔN-3Mut, the C-terminus α6 helix of PV 2C^ATPase^ was kept essentially straight in all 2C copies. We compared the PV 2C-2C conformation with multiple EV71 2C-2C conformations, which demonstrated that PV 2C-2C conformation resembles EV71 2C-2C conformation-2 [21], a catalytically nonproductive conformation. We measured the distance from the phosphate group occupying the P-loop of a PV-2C-ΔN-3Mut monomer to the side chain of R241 (arginine-finger) on an adjacent PV-2C-ΔN-3Mut. The distance was 21Å, suggesting this cannot be a catalytically active conformation. In order to explore the biologically relevant conformation of PV 2C^ATPase^, we built a hexameric ring model. We first searched structural homologues of PV-2C-ΔN-3Mut using Dali server, and found that beside EV71 2C, the best hit was the structure of a SV40 Large T Antigen (PDB code: 2H1L). We generated a hexameric model of PV 2C^ATPase^ by superimposing six copies of PV-2C-ΔN-3Mut to each subunit within SV40 Large T Antigen hexamer. In the hexameric model of PV-2C-ΔN, the C-terminal PBD of a protomer is located very close to the hydrophobic pocket on the adjacent protomer. We therefore slightly bent the α6 helix so that the PBD could readily occupy the hydrophobic pocket (Fig 5A).

The hexameric model of PV-2C-ΔN has a ring-like shape with the diameter of 117Å and the height of 40Å (Fig 5B). Its size is similar to EV71 2C hexameric model[21]. Adams and colleagues have reported that particles of the MBP tagged PV 2C^ATPase^ were composed of 5–8 protomers, and they exhibited increasing size ranging from 150 to 200Å [36]. The larger size of PV 2C^ATPase^ homo-oligomers visualized in the electron microcopy was possibly contributed by the N-terminal domain of PV 2C^ATPase^ and the presence of the MBP tag. While the unliganded foot-and-mouth disease virus 2C protein lacking the N-terminal domain self-oligomerizes in a concentration-dependent manner, this truncation containing a Motif C mutation (N207A) specifically forms hexamers in the presence of ATP and RNA[47]. The negative stain electron microscopy study further revealed FMDV 2C-ATP-RNA is a hexameric ring with 6-fold symmetry. The hexameric ring model of PV-2C-ΔN shows that all conserved ATPase motifs line up at the gaps between protomers, constituting the active sites (Fig 5A). While Walker A, Walker B motifs and Motif C are located on one side of the active site, the R finger (R241) is located on the other side. The distance from the phosphate group at the P-loop to R241 side chain has now reduced to 5.1Å, suggesting the improved active site conformation. The central pore of the hexameric ring has a funnel-like shape. While the opening on cytoplasm side is wider (diameter = 27Å), the opening on membrane proximal side is narrower (diameter = 14Å). It was previously reported that PV 2C^ATPase^ harbors two discrete segments, residues 21–45 and 312–319, both involved in RNA binding [10]. Although the N-terminal RNA binding segment is missing in our structure, the C-terminal RNA binding segment is located on the rim of the hexameric ring, suggesting that the RNA might bind the rim of PV 2C^ATPase^ hexameric ring.

**Fig 6:**
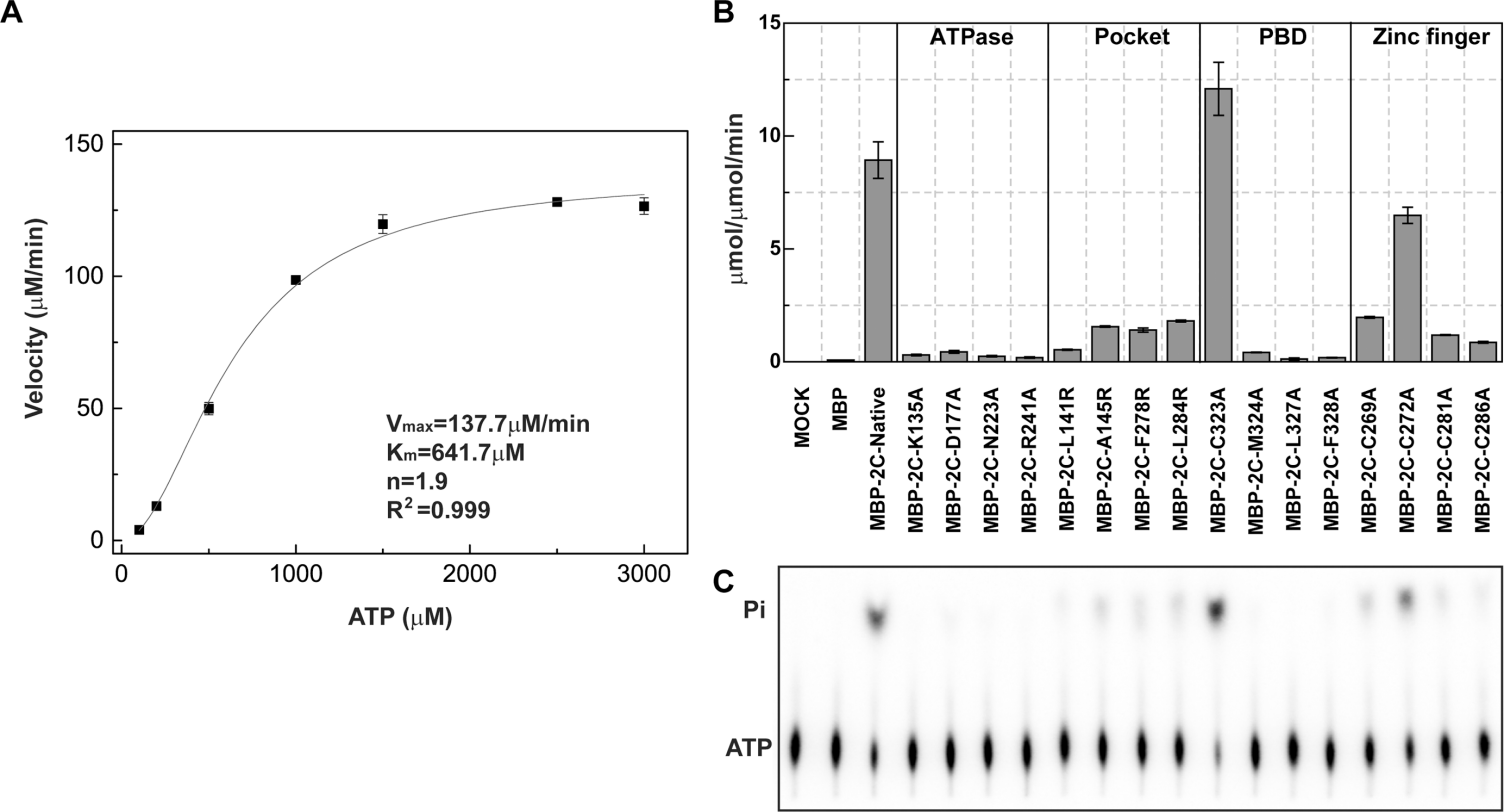
ATPase activities of various PV 2C mutants. (A) The velocity of ATP hydrolysis catalyzed by the MBP tagged PV 2C^ATPase^ (5μM) is plotted as the function of the substrate (ATP) concentration. The data was then fitted with the Hill equation with an R^2^ = 0.999. The kinetic parameters K_m_, V_max_ and the Hill coefficient “n” were calculated and indicated. (B) The ATP activity was measured for the MBP tagged PV 2C^ATPase^ and a selection of mutants. For each measurement, at least three time points were used to calculate the velocity of the reaction. The ATPase activity is expressed as how many μmol of ATP hydrolyzed per μmol of enzyme per minute. (C) An image of thin-layer chromatography plate of the ATP hydrolysis reaction visualized by Typhoon TrioVariable Mode Imager.

To validate our structural findings, we characterized the ATPase activity of the MBP tagged full-length PV 2C^ATPase^ and a selection of mutants. We first characterized the ATPase activity of the wild-type enzyme (Fig 6A). Our analysis showed that the ATPase activity of PV 2C^ATPase^ obeys the Hill equation. The nonlinear curve fitting using the Hill equation gave an R^2^ = 0.999. The Hill coefficient n is approximal 1.9, suggesting a positive cooperativity in ATP binding and hydrolysis. We calculated the enzyme kinetic parameters V_max_ = 137.7±2.1 μM/min and K_m_ = 641.7±25.1 μM. The turnover rate k_cat_ of the enzyme is 27.5 min^-1^. This value was calculated by dividing V_max_ with the concentration of the monomeric MBP-PV 2C. The turnover rate of the MBP-tagged PV 2C is lower than that measured for the bilayer nanodiscs bound PV 2C[48]. The difference in the ATPase activity is likely due to the different strategy and affinity tag used in protein preparation.

Next, we measured and compared the ATPase activity of the PV 2C^ATPase^ mutants (Fig 6B and 6C). When we kept the substrate ATP concentration constant at 500μM, the wild-type PV 2C^ATPase^ exhibited an ATPase activity of 8.9±0.8 μmol/μmol/min^-1^, whereas the catalytic inactive mutants K135A (Walker A), D177A (Walker B), N223A (Motif C) and R241A (R finger) all exhibited the background activities at least 20 folds less than the wild-type activity, similar to MOCK and MBP controls. In most of cases, mutations on either the PBD or the pocket led to severe losses or abrogation of ATPase activity. The loss of ATPase activity was possibly caused by the disruption of PBD-pocket interaction and in turn 2C self-oligomerization, therefore the active site between PV 2C-2C could not form. These results are consistent with size-exclusion chromatography experiment that the disruption of PBD-pocket interaction by mutation F328A or deletion of PBD abolished 2C tetramerization in solution (Fig 4). The ATPase activity of mutant C323A was measured as 12.1±1.2 μmol/μmol/min, similar to WT enzyme. This suggests that substituting of C323 by an alanine could not undermine the interaction between PBD-pocket. This hypothesis is supported by our size-exclusion chromatography results (Fig 4). PV-2C-ΔN bearing mutation C323A at the C-terminal PBD also eluted as tetramers, suggesting the homo-oligomerization remained unaffected (Fig 4).

**Fig 7:**
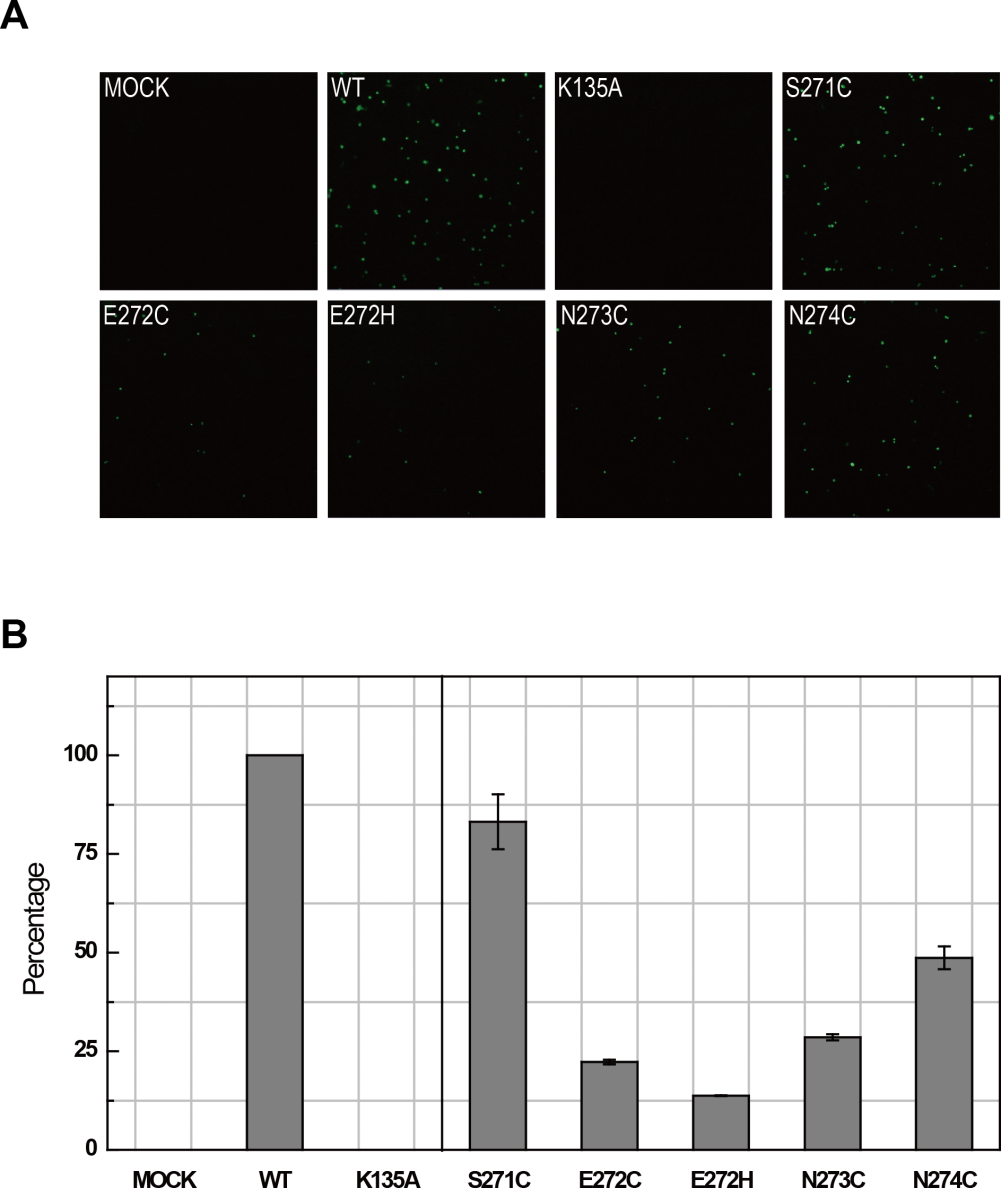
Role of PCS2 of the zinc finger of 2C in virus production. (A) Production of EV71 in revealed by immunofluorescence. Vero cells were transfected with RNA transcripts from an EV71 infectious clone bearing the indicated mutations. The mutant containing K135A (Walker A motif) was used as the negative control. Supernatant of the cell culture was used to infect RD cells. EV71 production was detected using anti-EV71 antibody and anti-mouse secondary antibody. (B) Histogram representation of the experiments shown in panel A. Production of the indicated mutants is quantified as percentage of the positive dots comparing to WT virus. The results are the mean value averaged from eight randomly selected visual fields.

Among alanine substitutions of four zinc coordinating cysteine, C272A retained more than 70% of WT ATPase activities, 6.5±0.4 μmol/μmol/min, whereas C269A, C281A and C286A lost the majority of their activities; the percentage of the activities remained were 22%, 13% and 10%, respectively. Pfister et al. showed that while eliminating PCS1 (C269) of PV 2C^ATPase^ caused the failure in recovering virus from plasmid, but eliminating PCS2 (substitute C272 with serine or glutamine) did not affected PV translation activity in vivo. Nevertheless, substitution of PCS2 could induce temperature sensitive phenotype, encapsidation defects and impairment of RNA replication at high temperature [43]. PCS2 is naturally missing in EV71 2C and many other enterovirus 2C proteins. To further investigate the significance of PCS2, we introduced a set of mutations to an infectious clone of EV71. Structural alignments of PV 2C^ATPase^ and EV71 2C (Fig 2D) revealed that residue E272 of EV71 2C is the structural counterpart of C272 of PV 2C^ATPase^. We therefore substituted E272 with cysteine and histidine respectively, with an aim of adding PCS2 to the EV71 2C zinc finger. To cover all possibilities, we also substituted three other nearby residues S271, N273 and N274 with cysteine respectively, so that at least one of these mutants may have a PCS2 for the zinc finger. To our surprises, the infectious clone bearing E272C or E272H did not showed the improved EV71 infectivity, instead they caused >75% losses of activities. Mutations N273C and N274C were also detrimental to virus production. Only S271C retained >75% of WT activity, but there was no improvement in EV71 production (Fig 7A and 7B).

## Discussion

Comparing the available high-resolution structures of enterovirus 2C proteins (PV 2C, EV-C species and EV71 2C, EV-A species), we identified both common and individual structural features. (i) The zinc finger is the most structurally distinct site in PV 2C^ATPase^ and EV71 2C. While PC 2C^ATPase^ has a CCCC type zinc finger, EV71 2C has only three cysteine (lacking the PCS2) for zinc coordination. Eliminating the PCS2 of PV 2C^ATPase^ resulted in temperature-sensitive phenotypes and encapsidation defects [11,49]. We added the PCS2 to the zinc finger of EV71 2C in order to convert it to a CCCC type zinc finger similar to PV 2C^ATPase^ zinc finger, but it failed to improve EV71 infectivity. Zinc fingers are ubiquitous small motifs that function as binding module for nucleic acids or proteins, etc.[50]. It was demonstrated that the C-terminal cysteine-rich site of PV 2C^ATPase^, the zinc finger in our structure, is required for morphogenesis[49]. Therefore, the significant difference in sequence and structure of the zinc finger we observed here may underlie the specificity of 2C protein and determine what process it may involve. The cysteine-rich motif is one of the least conserved regions in Picornaviridae 2C (Fig 2E). In fact, the 2C of the foot-and-mouth disease virus (FMDV) does not even have a cysteine-rich motif between ATPase and C-terminal helical domains. So, this region of FMDV 2C might fold into a structure completely different from the zinc finger but it may still function as protein binding module with the distinct specificity. (ii) The hexameric ring models of PV 2C^ATPase^ and EV71 2C show that the ATPase active site formed between 2C subunits has nearly identical geometry and the catalytic residues (Walker A & B, Motif C and R finger) identified by structural and biochemical characterizations are invariant. (iii) The C-terminus amphipathic helix mediated self-oligomerization is common in enterovirus 2C. Analogous to EV71 2C, PV 2C^ATPase^ undergoes self-oligomerization both in crystalline state and in solution via PBD-pocket interaction. Although residues constituting the PBD and the pocket are not strictly conserved in PV 2C^ATPase^ and EV71 2C, the “knob-into-hole” interaction between PBD-pocket is identical. The PBD residues of PV 2C^ATPase^ include C323, M324, L327 and F328, whereas the PBD of EV71 2C contains residues T323, I324, L327 and F328. All PBD residues are hydrophobic, in which L327 and F328 are invariant. The strict conservation of L327 and F328 highlights their essential role in 2C activity. We showed that mutations L327 and F328 abrogated the ATPase activity and homo-oligomerization of both PV 2C^ATPase^ and EV71 2C helicases (Figs 4 and 5), and these mutations could halt EV71 infection [21]. The dimension of the hydrophobic pocket of PV 2C^ATPase^ is 17Å long, 12 Å wide and 7Å deep, which is similar to the pocket size of EV71 2C. We calculated the solvent accessible surface area (SASA) of the pocket of PV 2C^ATPase^ as 918 Å^2^ containing 13 residues, the SASA of the pocket of EV71 2C is 846 Å^2^ containing 14 residues.

Eight zinc finger fold groups have been classified previously [50]. Comparison of the geometry of the zinc fingers of EV71 2C and PV 2C^ATPase^ with eight known groups of zinc finger demonstrates that it belongs to none of them (S3 Fig). Therefore, the 2C zinc finger represents a new fold group, which we denote the “Enterovirus 2C-like” fold group. This group is composed of a N-terminal long loop followed by a short helix and a short loop. Three zinc ligands are contributed from the long loop, one ligand is contributed from the short helix. The short helix has a “PhhC” consensus sequence (h represents hydrophobic residues) in enteroviruses. The PhhC sequence is followed by a “GKA” motif that is invariant among enteroviruses. The conserved lysine of GKA motif plays an auxiliary role in stabilizing the zinc binding. The second zinc ligand is nonessential in zinc coordination. It can be substituted by a solvent water molecule, such in case of EV71 2C [21].

**Fig 8:**
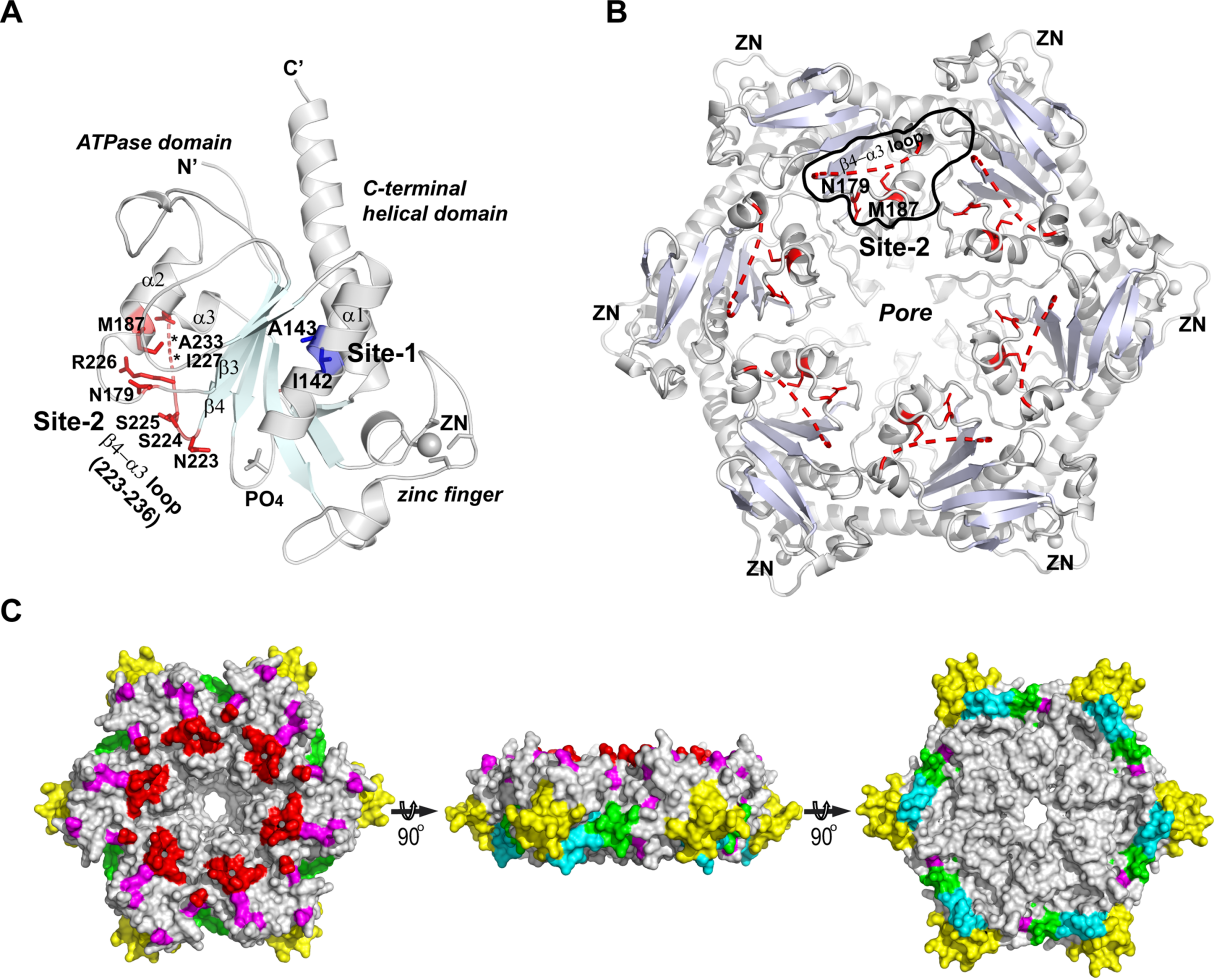
Visualizing key residues for 2C functions and drug resistant on structures. (A) Residues involved in GuHCl resistant are clustered in two distinct sites. Site1 (blue) is buried in the hydrophobic core of ATPase domain, site2 (red) is surface exposed. Residues of site1 and site2 are shown with stick model. (B) Site2 is located on the surface on cytoplasm side of PV-2C-ΔN hexameric ring. Site2 area is outlined on a 2C monomer on top of the ring. (C) Mapping of functionally important areas on molecular surface of PV-2C-ΔN hexameric ring (left, cytoplasm side, middle, rim of the ring; right, membrane proximal side). (1) red area: residues involved in drug resistant, clustered around central pore; (2) magenta area: residues important to VP3 interaction, uncoating, encapsidation morphogenesis and temperature-sensitive type, located near the zinc binding site; (3) yellow area: zinc finger, important to uncoating, encapsidation morphogenesis and temperature-sensitive; (4) green area: RNA binding; (5) cyan area: homo-oligomerization.

Previous studies have identified a large set of residues important to 2C functions. Genotype analyses of drug resistant virus clones have suggested many residues that were targeted by drugs. We summarized these findings and analyzed the compatibility of the published phenotypes with our structural characterization (S1 and S2 Tables and Fig 8). Based on their locations on 2C structure, we divided these residues into four categories. (i) Residues from Walker A motif, Walker B motif, Motif C and R finger are essential for ATPase/helicase activities. They are gathered in the gaps between subunits in the hexameric ring model of 2C protein. These residues are in general not involved in drug resistance, probably due to high genetic barrier. Only one GuHCl-resistant mutation was mapped to Walker A (A133T) of echovirus-9 2C [11]. (ii) Residues buried deeply into the hydrophobic core of 2C are important to overall folding. These include L125, V218, I142 A143, M246 and I248, most of which are buried between the parallel β-strand plane and the surrounding helices in ATPase domain. These residues were found important to encapsidation[31], morphogenesis [49] or temperature-sensitive virus phenotype [51]. Mutations of these residues account for resistant to GuHCl, MRL-1237 and Hydantoin. (iii) Residues exposed to the molecular surface of 2C hexamer model may directly interact with protein binding partners, RNA or drugs. Most of these residues are mapped on the cytoplasmic side and the rim of the ring, however a few are on the membrane proximal side (Fig 8). Therefore, in vivo the membrane proximal side of the hexameric ring model is unlikely to be the accessible molecular surface of 2C. During infection, this side should be attached to N-terminal portion of 2C, whose structure is yet to be determined. We further divided the surface exposed residues into different regions (Fig 8C). The majority of published drug resistant mutations were mapped into regions around the pore of the hexameric ring. These regions are potentially targeted by GuHCl, MRL-1237, HBB, TBZE-029, Fluoxetine, Dibucaine, Zuclopenthixol and Pirlindole. (iv) Residues located on N-terminal membrane binding domain are still missing in the available 2C structures.

GuHCl is among the earliest identified compounds effective on PV and other EVs. We mapped all published GuHCl-resistant or dependent mutations [9,10] on the structure of PV-2C-ΔN, revealing two distinct sites, site-1 and site-2 (Fig 8A and 8B). Mutations I142V and A143G cluster on site-1, where they are deeply buried inside the hydrophobic core of ATPase domain. Substitute at here may affect the interaction with GuHCl or other ligands via an indirect way; the mechanism require further investigation. The other mutations, N179G on the loop between β3 and α2, M187L on the α2 helix and S225T, I227M and A233T/S on the loop between β4 and α3 (β4-α3-loop), cluster on site-2. The β4-α3-loop is highly flexible in PV 2C^ATPase^, and a segment of this loop residues 227–235 were missing in the electron density map. When displayed these residues on the hexameric ring model of PV-2C-ΔN, we found that six copies of site-2 from each subunit form a belt region surrounding the central pore (Fig 8A and 8B), which may act as the direct binding site of GuHCl. Site-2 might act an essential interface for the binding with other viral or cellular proteins during replication. Hence, a plausible explanation of the inhibitory mechanism of GuHCl is blocking other proteins from binding to 2C by occupying site-2. Mutations responsible for resistance to other drugs were also mapped on the β4-α3-loop. Echovirus 9 bearing 2C mutations I227L and A229V is resistant to HBB. Coxsackievirus B3 containing 2C mutation A224V, I227V and A229V is resistant to TBZE-029, Fluoxetine and Dibucaine. Therefore, the β4-α3-loop of 2C is likely a “hot spot” for drug resistance.

The investigation of PV morphogenesis suggested that the cysteine-rich motif on the C-terminal domain of 2C helicase is involved in encapsidation, possibly via an interaction with a region between Walker A and B motifs of the ATPase domain [34,49]. Our crystal structure of PV 2C^ATPase^ revealed that the α1 helix (residues 135–150) located between the Walker A and B motifs (Fig 2E and 2C) directly interacts with residues N277 and F278 from the loop between PCS2 and PCS3 of the zinc finger. Wang et al showed that PV with K279A/R280A mutations showed defects in replication and encapsidation[49]. The same group later demonstrated that the PCSs of the cysteine-rich motif are also involved in encapsidation. According to the crystal structure of PV 2C^ATPase^ and the hexameric ring model, the cysteine rich motif folds into a zinc finger and it is located on six vertices of the ring (Fig 8C). K279 and R280 are located immediately downstream of N277 and F278, where they are fully accessible on the surface of the hexameric ring. Therefore, it is suggestive that the interaction between the zinc finger and the ATPase domains are important to stabilizing the folding of the zinc finger, whereas K279 and R280 may be involved in an interaction with other protein partners during encapsidation. Among four PCSs of the zinc finger, C269 (PCS1) and C281 (PCS3) are almost completely buried in the hydrophobic core; by contrast, C272 (PCS2) and C286 (PCS4) are relatively more exposed to the molecular surface. This supports the results reported by Wang and colleagues [49], that while alanine substitution of PCS1 or PCS3 was lethal, substitutions of PCS4 and PCS2 exhibited temperature-sensitive and quasi infectious phenotypes respectively. It suggests that PCS1 and PCS3 are absolutely required in maintaining the folding of the zinc finger and probably the entire hexamer, whereas PCS2 and PCS4 might have an additional role of interacting with other proteins during encapsidation.

## Material and methods

### Cells and reagents

Vero and RD cells (American Type Culture Collection) were grown in Dulbecco’s modified Eagle’s medium (DMEM, Gibco) with 10% fetal bovine serum added. EV71 infectious clone was a gift from S. Cen (Institute of Medicinal Biotechnology, Chinese Academy of Medical Sciences and Peking Union Medical College) which contained the full-length cDNA of WT EV71 (strain Fuyang, GenBank: EU703814.1). The mouse anti-EV71 monoclonal antibody was purchased from Millipore. Donkey anti-mouse immunoglobulin G secondary antibody was purchased from LI-COR Biosciences.

### Protein preparation

Because full-length PV 2C^ATPase^is unstable for structural and biochemical studies, we prepared the maltose-binding protein (MBP) tagged 2C^ATPase^ as described previously [36]. In brief, the cDNA encoding 2C^ATPase^ of human poliovirus 1 strain Mahoney (GenBank ID: KU866422.1) was synthesized (GENEWIZ), amplified using PCR and inserted into pMAL-c5X vector (New England BioLabs) between EcoRV and SalI sites. The resulting plasmid expressed a N-terminal tagged full-length PV 2C^ATPase^ (MBP-PV 2C). The plasmid was transformed to competent E. coli cells BL21 (DE3). The bacteria were inoculated in a medium containing 100 mg/L ampicillin and grown to a density OD_600_ = 0.8 at 37°C. The cultures were cooled and induced with 0.3mM IPTG. The bacteria were then cultured at 18°C overnight before harvesting. The cells were pelleted by centrifugation (5,000g, 10min) and resuspended in a cold lysis buffer (50mM HEPES pH 7.5, 500mM NaCl, 1% Triton X-100, 1mM DTT and 1mM EDTA) on ice. The suspended cells were disrupted by ultrasonication on ice and clarified by centrifugation at 25,000g for 30min. The supernatant was loaded onto the amylose resin (New England BioLabs) and the resin was washed thoroughly by using a buffer containing 50mM HEPES pH 7.5, 500mM NaCl, 1% Triton X-100 and 1mM DTT. The MBP-PV 2C protein was eluted with 5 column volume of elution buffer (50mM HEPES, pH 7.5, 100mM NaCl, 1mM DTT and 20mM maltose). The final step of the purification was size exclusion chromatography using Superdex 200 HR10/300 GL column (GE Healthcare) pre-equilibrated with a buffer containing 20mM HEPES pH 7.5 and 100mM NaCl and 5mM DTT.

The MBP tagged full-length PV 2C^ATPase^ were used for biochemical characterization, however, this protein did not yield crystals. To crystallized PV 2C^ATPase^, we expressed a fragment of 2C lacking the N-terminal membrane binding domain. The cDNA encoding residues 116–329 of PV 2C^ATPase^ was amplified PCR and inserted into pET28-SUMO expression plasmid [21](modified from pET28a) between BglII and XhoI sites. The resulting plasmid expressed a truncation of PV 2C^ATPase^, denoted PV-2C-ΔN. The plasmid was transformed into E. coli competent cells BL21 (DE3) (Novagen). A single colony was picked and grew in 1L of LB media containing 50 mg/L kanamycin at 37°C to a density OD_600_ = 0.8. The culture was then cooled to 18°C and induced by 0.5mM IPTG (final concentration). The bacteria culture continued at 18°C overnight. The cells were harvested by centrifugation (5,000g, 10min) and resuspended in cold lysis buffer (50mM Tris-HCl pH 7.5 and 100mM NaCl) and disrupted by ultrasonication. The cell debris was removed by centrifugation at 25,000g for 30 min. The supernatant was loaded to Ni-NTA resin (Qiagen). The column was washed thoroughly with 20 column volumes of wash buffer containing 50mM Tris pH 7.5, 100mM NaCl, 20mM imidazole to eliminate nonspecifically bound proteins. Subsequently, Ulp1 peptidase was added to cleave SUMO tag at 4°C overnight. The flow through containing the non-tagged PV-2C-ΔN was collected and subjected to Superdex 200 HR10/300 GL column (GE Healthcare) pre-equilibrated with a buffer containing 20mM HEPES pH 7.5 and 100mM NaCl and 5mM DTT.

Mutations of the full-length MBP-PV 2C^ATPase^ and PV-2C-ΔN were introduced by site-directed mutagenesis. The mutants were expressed and purified using the same protocols described above.

### Crystallization and structure determination

PV-2C-ΔN-3Mut was concentrated to ~5 mg/ml before crystallization trials. The protein was crystallized by mixing 1μl of protein sample (5mg/ml) with 1.3μl of buffer containing 0.2M MgCl_2_, 0.1M MES pH 6.5, 3% (v/v) PEG4000, 9.2% (v/v) polypropylene glycol P 400, and 5mM TECP were freshly added to the buffer before use. The crystals were grown in a hanging-drop vapor diffusion system at 20°C. The crystals were flash frozen in Liquid nitrogen. Glycerol (v/v 25%) was used as the cryoprotectant. Complete datasets were collected at BL18U1 beamline of Shanghai Synchrotron Radiation Facility (SSRF). The crystal diffracted the X ray to 2.55Å. It belonged to a space group of P2_1_, contained six copies of PV 2C-ΔN in the asymmetric unit (ASU). The structure was solved by molecular replacement using EV71 2C structure (PDB code: 5GQ1, B chain) as the searching model. The atomic model of PV-2C-ΔN-3Mut was completed by manual building using the software Coot v0.8.2 [52]. The structure was refined using the software PHENIX v1.10.1[53]. Most residues of PV-2C-ΔN-3Mut were located in chain A, B, C, D and E, however only a fraction of residues was visible in chain H. The loop between β4 and α3 was highly flexible in all chains. The composite omit map of PV-2C-ΔN-3Mut was calculated using the software phenix.composite_omit_map from PHENIX v1.10.1[53]. The software was run in a simulated annealing mode to aggressively remove phase bias. The annealing method used was cartesian and the starting temperature was 5,000 K. The statistics of data collection, structure refinement and structure validation were summarized in Table 1.

### ATPase assay

ATPase assays were performed as previously described [21,36,54]. The concentration of the enzyme was kept constant at 5μM in all reactions. The volume of the reaction mixtures was 50μl, contained 20mM HEPES pH 7.5, 4mM magnesium acetate, 5mM DTT, 500μM ATP and trace amount of [γ-^32^P] labelled ATP. The mixtures were incubated at 30°C and the reactions were initiated by adding enzyme. At the given time point, 10μL of the reaction mixture was removed and mixed with EDTA (final concentration = 0.1M) to quench the reaction. At least three time points were recorded for each reaction. The mixtures were resolved by thin-layer chromatography using PEI (Polyethylenimine) Cellulose Plates (Sigma-Aldrich) with a buffer containing 0.8M acetic acid and 0.8M Lithium chloride [21]. The PEI plates were visualized and quantified using Typhoon TrioVariable Mode Imager (GE Healthcare).

### EV71 production efficiency from infectious clones

Vero and RD cells were grown with 5% CO_2_ in DMEM, supplemented with 10% Fetal Bovine serum. EV71 infectious clone contained the full-length cDNA of WT virus. The mutations were introduced by the site-directed mutagenesis. The plasmids were linearized by HindIII digestion and were in vitro transcribed to RNAs using the MEGA script T7 Kit (Ambion). Subsequently, the RNAs were transfected in Vero cells with Lipofectamine 2000 (Invitrogen) according to manufacturer’s instructions. The Vero cells were cultured at 37°C. 72 hours post-transfection, the supernatants of cell culture were collected to infect RD cells seeded in a 24-well plate at the given temperatures. The RD cells were fixed 24 hours post-infection. Immunofluorescence assays were performed to probe the production of EV71 virus using mouse anti-EV71 monoclonal antibody and Donkey anti-mouse immunoglobulin G secondary antibody. At least eight different visual fields from each well were photographed randomly under the microscope. The positive dots were counted using the software ImageJ v1.2.0 [55].

### Size-exclusion chromatography

The Superdex200 10/300 GL column (GE Healthcare) was equilibrated with a buffer containing 20mM HEPES pH 7.5 and 100mM NaCl and 5mM DTT. The column was calibrated with Gel Filtration Standard (BIO-RAD) containing thyroglobulin (670kDa), γ-globulin (158kDa), ovalbumin (45kDa), myoglobin (17kDa), and vitamin B12 (1.35kDa). The purified proteins were loaded and eluted with a flow rate of 0.5ml/min. The Log of the molecular mass (kDa) of the standards was plotted as the function of the elution volume. Four standard proteins: thyroglobulin, γ-globulin, ovalbumin and myoglobin were used for a linear-fitting to generate a standard curve. The molecular mass of PV-2C-ΔN variants was then calculated using the standard curve.

## Supporting information

S1 Fig Structural comparison of different chains in the crystal structure of PV-2C-ΔN-3Mut. The ribbon models of chain A (red), chain B (orange), chain C (yellow), chain D (green), chain E (cyan) and chain H (blue) were aligned using Dali pairwise structure comparison server. The structural comparison between chains A and B gave Z-score = 33.1, rmsd = 1.3Å with 204 residues aligned. The largest structural deviation between A and B chains is contributed by a small loop region 180-184aa of B chain between β3 and α2, which is resulted from the crystal packing. The structural comparison between chains A and C gave Z-score = 31.5, rmsd = 0.5Å with 188 residues aligned. The structural comparison between chains A and D gave Z-score = 34.6, rmsd = 0.4 Å with 202 residues aligned. The structural comparison between chains A and E gave Z-score = 32.8, rmsd = 0.8Å with 201 residues aligned. The structural comparison between chains A and H gave Z-score = 10, rmsd = 1.2Å with 89 residues aligned. The C-terminal helix of different chains exhibits only slightly different conformation, but this is not comparable with the large conformational changes observed at the C-terminal helix in EV71 2C structure (PDB entries: 5GQ1 and 5GRB).
(DOCX)

S2 Fig CD spectra of the MBP-tagged PV 2C and a mutant bearing mutations E207A, K209A and R149A. The CD spectrum of the MBP-tagged PV 2C with the wild-type sequence (red) is overlaid with the CD spectrum of the mutant (E207A, K209A and R149A) used for crystallization. The experiment shows that the triple mutations does not affect the overall folding of the protein.
(DOCX)

S3 Fig The zinc finger of enterovirus 2C proteins belongs to a new fold group. epresentative structures of different zinc fingers. The structures are shown with ribbon models and colored by secondary structure elements; α-helices are red, β-sheets are yellow, loops are green. The zinc ions are shown with gray spheres. Ligands for zincs, histidine or cysteine, are shown with stick models. (A) Left, structure of PV 2C zinc finger with 4 cysteine ligands (Cys4 type); right structure of EV71 2C zinc finger with 3 cysteine ligands (Cys3 type). These zinc fingers cannot be placed into any of the eight known zinc finger fold groups (illustrated in panel B-I), therefore, we classify them into a new fold group, denoted, Enterovirus 2C-like group. B to I. Representative structures from eight zinc finger fold groups defined by Krishna and colleagues. (B) Fold group 1: C2H2-like, PDB code: 1T6D chain D residues 42–69. (C) Fold group 2: Gag knuckle, PDB code: 1A1T chain A residues 12–30. (D) Fold group 3: treble clef, PDB code: 1HCQ chain A residues 5–36. (E) Fold group 4: zinc ribbon, PDB code: 1TFI chain A residues 1–50. (F) Fold group 5: Zn2/Cys6 like, PDB code: 2HAP chain C residues 62–95. (G) Fold group 6: TAZ2 domain like, PDB code: 1F81 chain A residues 37–65. (H) Fold group 7: zinc binding loops, PDB code: 1I3Q chain C residues 84–96. (I) Fold group 8: metallothioneins, PDB code: 4MT2 chain A residues 1–61.
(DOCX)

S1 Table Previous molecular genetic studies of 2C proteins and the compatibility with known 2C structures. (DOC)

S2 Table Drug resistant mutations and the compatibility with 2C structures. (DOC)

S1 File The PDB X-ray structure validation report. (PDF)
