# Supplementary material for: Crystal structure of a soluble fragment of poliovirus 2CATPase
Source: PLoS Pathog. 2018 Sep 19;14(9):e1007304. doi: 10.1371/journal.ppat.1007304 (PMC6166989; doi:10.1371/journal.ppat.1007304)
Supplement: S1 Fig — The ribbon models of chain A (red), chain B (orange), chain C (yellow), chain D (green), chain E (cyan) and chain H (blue) were aligned using Dali pairwise structure comparison server. The structural comparison between chains A and B gave Z-score = 33.1, rmsd = 1.3Å with 204 residues aligned. The largest structural deviation between A and B chains is contributed by a small loop region 180-184aa of B chain between β3 and α2, which is resulted from the crystal packing. The structural comparison between chains A and C gave Z-score = 31.5, rmsd = 0.5Å with 188 residues aligned. The structural comparison between chains A and D gave Z-score = 34.6, rmsd = 0.4 Å with 202 residues aligned. The structural comparison between chains A and E gave Z-score = 32.8, rmsd = 0.8Å with 201 residues aligned. The structural comparison between chains A and H gave Z-score = 10, rmsd = 1.2Å with 89 residues aligned. The C-terminal helix of different chains exhibits only slightly different conformation, but this is not comparable with the large conformational changes observed at the C-terminal helix in EV71 2C structure (PDB entries: 5GQ1 and 5GRB). (DOCX) [file ppat.1007304.s001.docx]

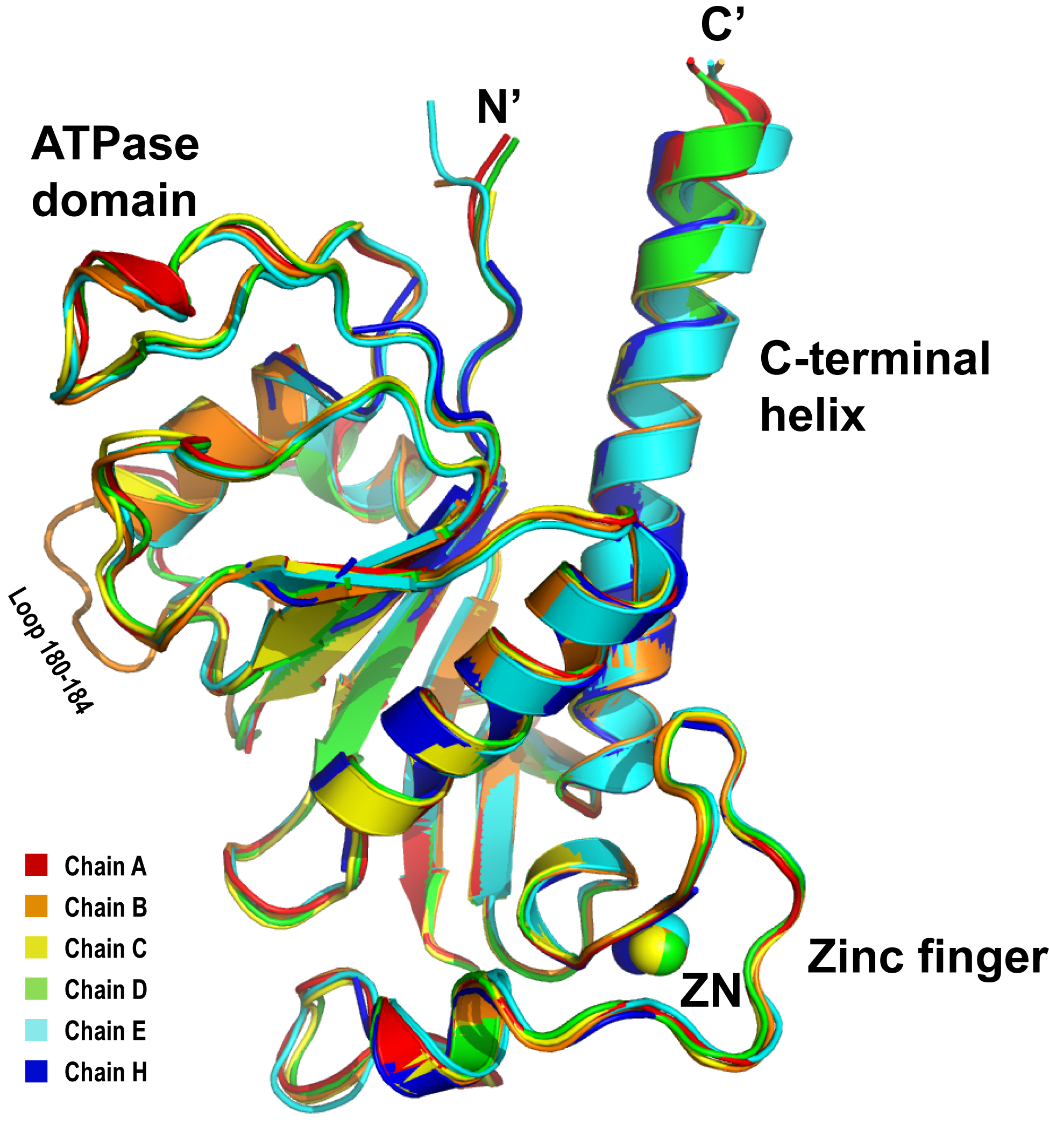


**Fig S1. Structural comparison of different chains in the crystal structure of PV 2C ΔN.**

The ribbon models of chain A (red), chain B (orange), chain C (yellow), chain D (green), chain E (cyan) and chain H (blue) were aligned using Dali pairwise structure comparison server. The structural comparison between chains A and B gave Z-score=33.1, rmsd=1.3Å with 204 residues aligned. The largest structural deviation between A and B chains is contributed by a small loop region 180-184aa of B chain between β3 and α2, which is resulted from the crystal packing. The structural comparison between chains A and C gave Z-score=31.5, rmsd=0.5Å with 188 residues aligned. The structural comparison between chains A and D gave Z-score=34.6, rmsd=0.4 Å with 202 residues aligned. The structural comparison between chains A and E gave Z-score=32.8, rmsd=0.8Å with 201 residues aligned. The structural comparison between chains A and H gave Z-score=10, rmsd=1.2Å with 89 residues aligned. The C-terminal helix of different chains exhibits only slightly different conformation, but this is not comparable with the large conformational changes observed at the C-terminal helix in EV71 2C structure (PDB entries: 5GQ1 and 5GRB).
