# Supplementary material for: Crystal structure of a soluble fragment of poliovirus 2CATPase
Source: PLoS Pathog. 2018 Sep 19;14(9):e1007304. doi: 10.1371/journal.ppat.1007304 (PMC6166989; doi:10.1371/journal.ppat.1007304)
Supplement: S2 Fig — The CD spectrum of the MBP-tagged PV 2C with the wild-type sequence (red) is overlaid with the CD spectrum of the mutant (E207A, K209A and R149A) used for crystallization. The experiment shows that the triple mutations does not affect the overall folding of the protein. (DOCX) [file ppat.1007304.s002.docx]

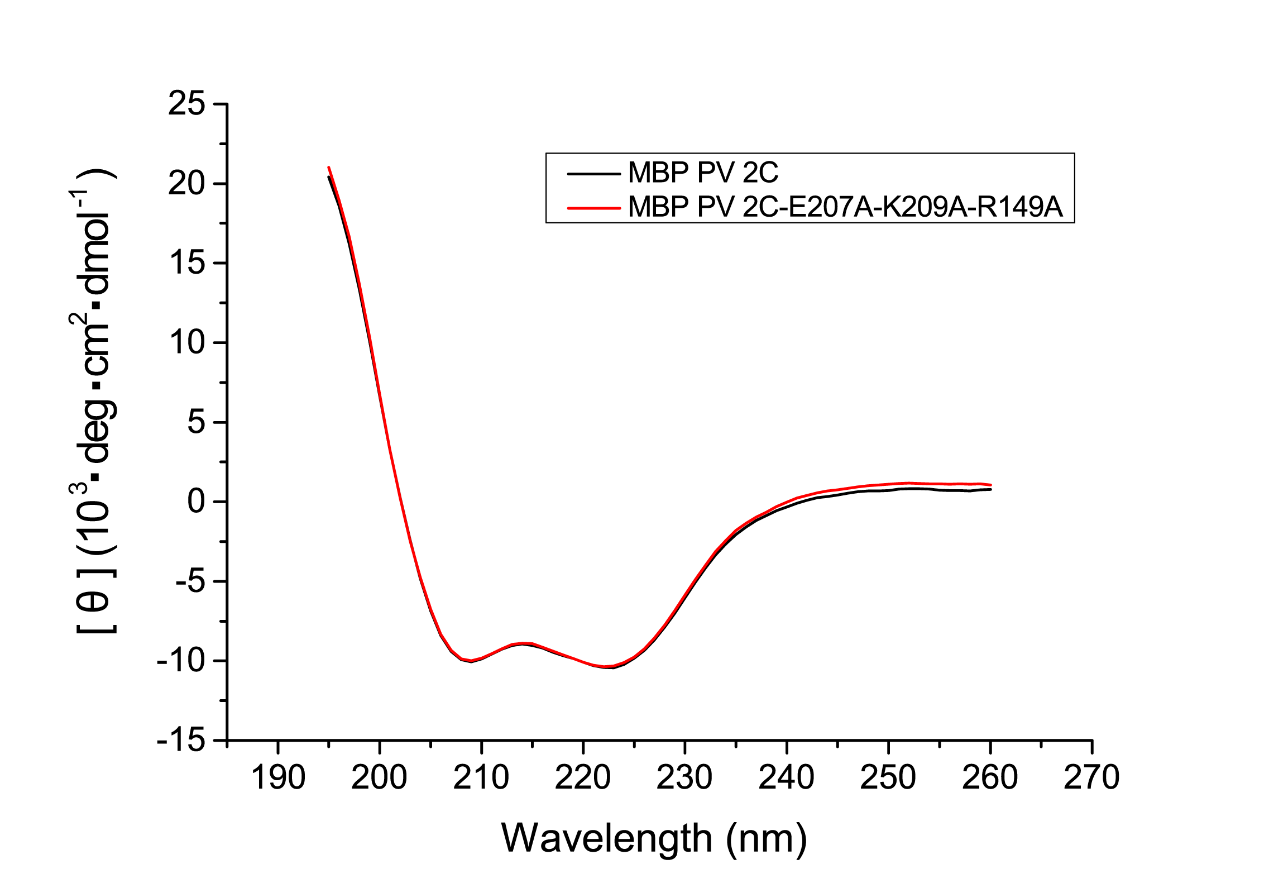


**Fig S2. CD spectra of the MBP-tagged PV 2C and a mutant bearing mutations** **E207A, K209A and R149A.**

The CD spectrum of the MBP-tagged PV 2C with the wild-type sequence (red) is overlaid with the CD spectrum of the mutant (E207A, K209A and R149A) used for crystallization. The experiment shows that the triple mutations does not affect the overall folding of the protein.
