# Supplementary material for: Crystal structure of a soluble fragment of poliovirus 2CATPase
Source: PLoS Pathog. 2018 Sep 19;14(9):e1007304. doi: 10.1371/journal.ppat.1007304 (PMC6166989; doi:10.1371/journal.ppat.1007304)
Supplement: S3 Fig — epresentative structures of different zinc fingers. The structures are shown with ribbon models and colored by secondary structure elements; α-helices are red, β-sheets are yellow, loops are green. The zinc ions are shown with gray spheres. Ligands for zincs, histidine or cysteine, are shown with stick models. (A) Left, structure of PV 2C zinc finger with 4 cysteine ligands (Cys4 type); right structure of EV71 2C zinc finger with 3 cysteine ligands (Cys3 type). These zinc fingers cannot be placed into any of the eight known zinc finger fold groups (illustrated in panel B-I), therefore, we classify them into a new fold group, denoted, Enterovirus 2C-like group. B to I. Representative structures from eight zinc finger fold groups defined by Krishna and colleagues. (B) Fold group 1: C2H2-like, PDB code: 1T6D chain D residues 42–69. (C) Fold group 2: Gag knuckle, PDB code: 1A1T chain A residues 12–30. (D) Fold group 3: treble clef, PDB code: 1HCQ chain A residues 5–36. (E) Fold group 4: zinc ribbon, PDB code: 1TFI chain A residues 1–50. (F) Fold group 5: Zn2/Cys6 like, PDB code: 2HAP chain C residues 62–95. (G) Fold group 6: TAZ2 domain like, PDB code: 1F81 chain A residues 37–65. (H) Fold group 7: zinc binding loops, PDB code: 1I3Q chain C residues 84–96. (I) Fold group 8: metallothioneins, PDB code: 4MT2 chain A residues 1–61. (DOCX) [file ppat.1007304.s003.docx]

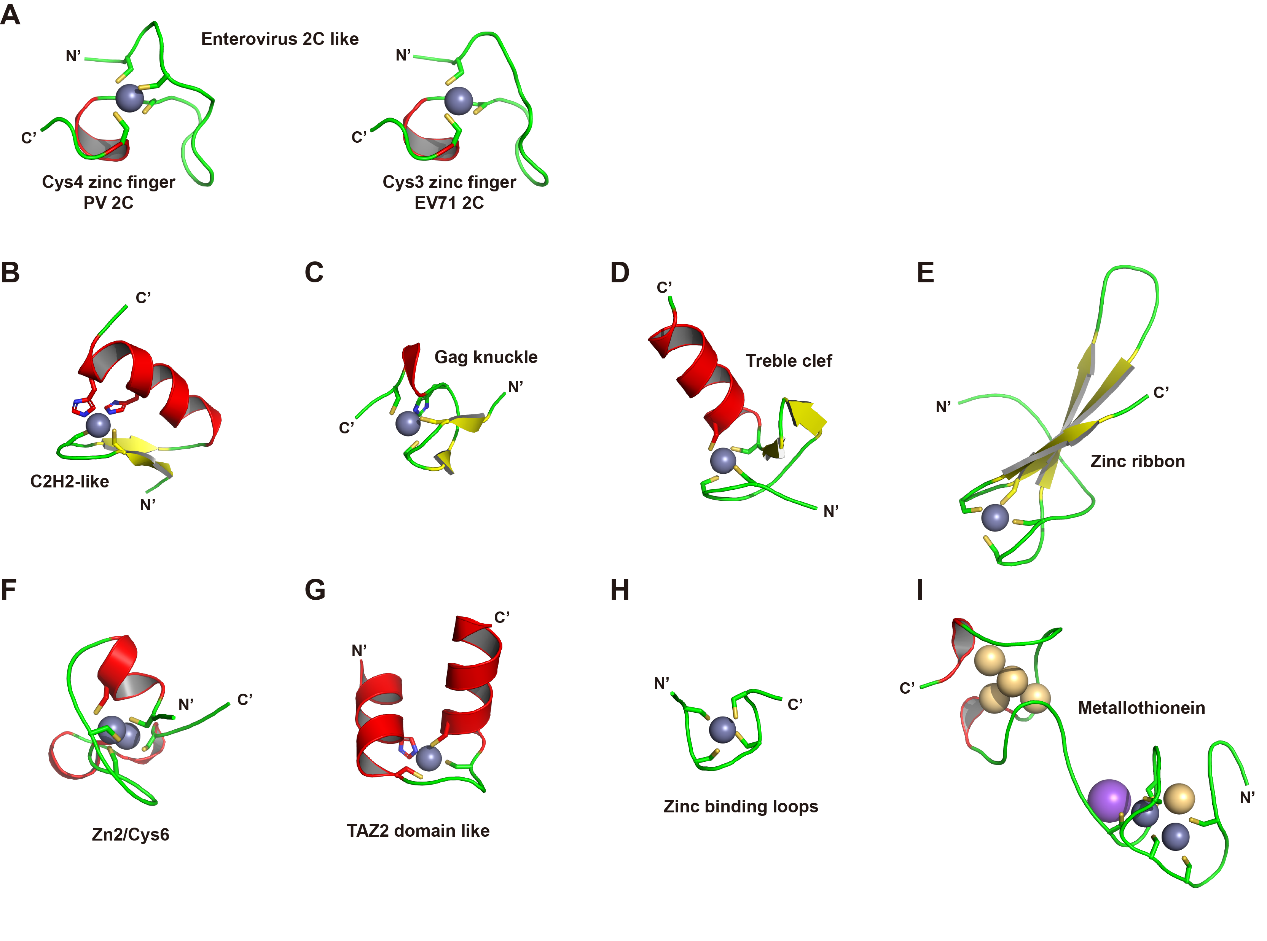


**S3 Fig. The zinc finger of enterovirus 2C helicases belongs to a new fold group** Representative structures of different zinc fingers. The structures are shown with ribbon models and colored by secondary structure elements; α-helices are red, β-sheets are yellow, loops are green. The zinc ions are shown with gray spheres. Ligands for zincs, histidine or cysteine, are shown with stick models. (A) Left, structure of PV 2C zinc finger with 4 cysteine ligands (Cys4 type); right structure of EV71 2C zinc finger with 3 cysteine ligands (Cys3 type). These zinc fingers cannot be placed into any of the eight known zinc finger fold groups (illustrated in panel B-I), therefore, we classify them into a new fold group, denoted, Enterovirus 2C-like group. B to I. Representative structures from eight zinc finger fold groups defined by Krishna and colleagues. (B) Fold group 1: C2H2-like, PDB code: 1T6D chain D residues 42-69. (C) Fold group 2: Gag knuckle, PDB code: 1A1T chain A residues 12-30. (D) Fold group 3: treble clef, PDB code: 1HCQ chain A residues 5-36. (E) Fold group 4: zinc ribbon, PDB code: 1TFI chain A residues 1-50. (F) Fold group 5: Zn2/Cys6 like, PDB code: 2HAP chain C residues 62-95. (G) Fold group 6: TAZ2 domain like, PDB code: 1F81 chain A residues 37-65. (H) Fold group 7: zinc binding loops, PDB code: 1I3Q chain C residues 84-96. (I) Fold group 8: metallothioneins, PDB code: 4MT2 chain A residues 1-61.
