# Supplementary material for: Crystal structure of a soluble fragment of poliovirus 2CATPase
Source: PLoS Pathog. 2018 Sep 19;14(9):e1007304. doi: 10.1371/journal.ppat.1007304 (PMC6166989; doi:10.1371/journal.ppat.1007304)
Supplement: S1 Table — (DOC) [file ppat.1007304.s004.doc]

**S1 Table. Previous molecular genetic studies of 2C helicases and the compatibility with known 2C structures.**

| ***Functions*** | ***Virus tested*** | ***Residues or regions*** | ***References*** | ***Location on structure*** |
| --- | --- | --- | --- | --- |
| RNA binding | PV1 | 21-45;  312-319; |  | Missing;  On the rim of hexameric ring; |
| ATP binding | PV1 | 129-136; |  | Walker A motif of the active site, between 2C-2C in hexameric ring; |
| Mg^2+^ binding | PV1 | 172-177; |  | Walker B motif of the active site, between 2C-2C in hexameric ring; |
| ATPase/Helicase activity | EV71 | 217-223; |  | Motif C of the active site, between 2C-2C in hexameric ring; |
| ATPase/Helicase activity | PV1, EV71 | R241; |  | R finger, between 2C-2C in hexameric ring; |
| VP3 binding | PV1, | N252; |  | On cytoplasm side of hexameric ring, near zinc finger domain; |
| Zinc binding | PV1 | 269-286; |  | Zinc finger domain; |
| Membrane binding | PV1 | 21-54; |  | Missing; |
| Self-oligomerization | PV1, EV71 | 1-38;  320-329; |  | Missing;  On the rim of hexameric ring; |
| Encapsidation | PV1 | K279/R280, C272, H273;  N252,K259;  Q65;  L125, V218; |  | Zinc finger domain;  On cytoplasm side of hexameric ring, near zinc finger;  Missing;  Buried in the hydrophobic core; |
| Uncoating | PV1 | K259, M293, K295;  C272, H273; |  | On cytoplasm side of hexameric ring, near zinc finger;  Zinc finger domain; |
| Temperature-sensitive | PV1 | C272, H273;  E150;  M246, I248； |  | Zinc finger domain;  On membrane proximal side of hexameric ring, near zinc finger domain;  Buried in the hydrophobic core; |
| Cold-sensitive | PV1, CVA20 | K259; |  | On cytoplasm side of hexameric ring, near zinc finger; |
| Cold-adapted | PV1 | V218;  R241;  M309; |  | Buried in the hydrophobic core;  R finger of the active site;  On the rim of hexameric ring |
| Morphogenesis | PV1 | Q65;  L125, V218;  K279, R280; |  | Missing;  Buried in the hydrophobic core;  Zinc finger domain; |

**References**

1. Tolskaya EA, Romanova LI, Kolesnikova MS, Gmyl AP, Gorbalenya AE, Agol VI. Genetic studies on the poliovirus 2C protein, an NTPase. A plausible mechanism of guanidine effect on the 2C function and evidence for the importance of 2C oligomerization. Journal of molecular biology. 1994;236(5):1310-23. Epub 1994/03/11. PubMed PMID: 8126722.

2. Wang C, Ma HC, Wimmer E, Jiang P, Paul AV. A C-terminal, cysteine-rich site in poliovirus 2C(ATPase) is required for morphogenesis. The Journal of general virology. 2014;95(Pt 6):1255-65. Epub 2014/02/22. doi: 10.1099/vir.0.062497-0. PubMed PMID: 24558221; PubMed Central PMCID: PMCPmc4027037.

3. Semler BL, Xia H, Wang P, Wang G-C, Yang J, Sun X, et al. Human Enterovirus Nonstructural Protein 2CATPase Functions as Both an RNA Helicase and ATP-Independent RNA Chaperone. PLoS pathogens. 2015;11(7):e1005067. doi: 10.1371/journal.ppat.1005067.

4. Guan H, Tian J, Qin B, Wojdyla JA, Wang B, Zhao Z, et al. Crystal structure of 2C helicase from enterovirus 71. 2017;3(4):e1602573. doi: 10.1126/sciadv.1602573. PubMed PMID: 28508043.

5. Liu Y, Wang C, Mueller S, Paul AV, Wimmer E, Jiang P. Direct interaction between two viral proteins, the nonstructural protein 2C and the capsid protein VP3, is required for enterovirus morphogenesis. PLoS pathogens. 2010;6(8):e1001066. Epub 2010/09/25. doi: 10.1371/journal.ppat.1001066. PubMed PMID: 20865167; PubMed Central PMCID: PMCPmc2928791.

6. Klein M, Hadaschik D, Zimmermann H, Eggers HJ, Nelsen-Salz B. The picornavirus replication inhibitors HBB and guanidine in the echovirus-9 system: the significance of viral protein 2C. The Journal of general virology. 2000;81(Pt 4):895-901. Epub 2000/03/22. doi: 10.1099/0022-1317-81-4-895. PubMed PMID: 10725414.

7. Echeverri AC, Dasgupta A. Amino terminal regions of poliovirus 2C protein mediate membrane binding. Virology. 1995;208(2):540-53. Epub 1995/04/20. doi: 10.1006/viro.1995.1185. PubMed PMID: 7747426.

8. Adams P, Kandiah E, Effantin G, Steven AC, Ehrenfeld E. Poliovirus 2C Protein Forms Homo-oligomeric Structures Required for ATPase Activity. Journal of Biological Chemistry. 2009;284(33):22012-21. doi: 10.1074/jbc.M109.031807.

9. Wang C, Jiang P, Sand C, Paul AV, Wimmer E. Alanine scanning of poliovirus 2CATPase reveals new genetic evidence that capsid protein/2CATPase interactions are essential for morphogenesis. J Virol. 2012;86(18):9964-75. Epub 2012/07/05. doi: 10.1128/jvi.00914-12. PubMed PMID: 22761387; PubMed Central PMCID: PMCPmc3446611.

10. Asare E, Mugavero J, Jiang P, Wimmer E, Paul AV, Sandri-Goldin RM. A Single Amino Acid Substitution in Poliovirus Nonstructural Protein 2CATPaseCauses Conditional Defects in Encapsidation and Uncoating. Journal of Virology. 2016;90(14):6174-86. doi: 10.1128/jvi.02877-15.

11. Vance LM, Moscufo N, Chow M, Heinz BA. Poliovirus 2C region functions during encapsidation of viral RNA. J Virol. 1997;71(11):8759-65. Epub 1997/10/29. PubMed PMID: 9343235; PubMed Central PMCID: PMCPmc192341.

12. <1990 An Intragenic Revertant of a Poliovirus 2C Mutant Has an Uncoating Defect.pdf>.

13. Li JP, Baltimore D. An intragenic revertant of a poliovirus 2C mutant has an uncoating defect. J Virol. 1990;64(3):1102-7. Epub 1990/03/01. PubMed PMID: 2154595; PubMed Central PMCID: PMCPmc249223.

14. Li JP, Baltimore D. Isolation of poliovirus 2C mutants defective in viral RNA synthesis. J Virol. 1988;62(11):4016-21. Epub 1988/11/01. PubMed PMID: 2845120; PubMed Central PMCID: PMCPmc253830.

15. Dove AW, Racaniello VR. Cold-adapted poliovirus mutants bypass a postentry replication block. J Virol. 1997;71(6):4728-35. Epub 1997/06/01. PubMed PMID: 9151866; PubMed Central PMCID: PMCPmc191694.
