# Supplementary material for: Crystal structure of a soluble fragment of poliovirus 2CATPase
Source: PLoS Pathog. 2018 Sep 19;14(9):e1007304. doi: 10.1371/journal.ppat.1007304 (PMC6166989; doi:10.1371/journal.ppat.1007304)
Supplement: S2 Table — (DOC) [file ppat.1007304.s005.doc]

**S2 Table. Drug resistant mutations and the compatibility with 2C structures**

| ***Inhibitors*** | | ***Virus*** | | Drug***-resistant mutations*** | | ***References*** | | ***Location on structure*** |
| --- | --- | --- | --- | --- | --- | --- | --- | --- |
|  | | PV1 | | I142V, A143G;  N179G, M187L, S225T, I227M, A233T/S; | |  | | Buried in the hydrophobic core;  On a solvent accessible area on the cytoplasm side of hexameric ring, around the central pore; |
| GuHCl | ECHOV9 | | E64G;  A133T; | |  | | Missing;  Walker A motif of the active site; | |
|  | CVB3 | | A224V | |  | | On the cytoplasm side of hexameric ring model, around the central pore; | |
| MRL-1237 | PV1  CVB3 | | I120V, F164Y;  N179A/G, I227V; | |  | | Buried in the hydrophobic core;  On a solvent accessible area on the cytoplasm side of hexameric ring, around the central pore; | |
| Hydantoin | PV1 | | Q65R;  L125V, V218I,V194I;  H118Y; | |  | | Missing;  Buried in the hydrophobic core;  On membrane proximal side of hexameric ring; | |
| BFA | PV1 | | V80I; | |  | | Missing; | |
| HBB | ECHOV9 | | I227L, A229V | |  | | On a solvent accessible area on the cytoplasm side of hexameric ring, around the central pore; | |
| TBZE-029 | CVB3 | | A224V, I227V, A229V; | |  | | On a solvent accessible area on the cytoplasm side of hexameric ring, around the central pore; | |
| Fluoxetine | CVB3 | | A224V, I227V, A229V; | |  | | On a solvent accessible area on the cytoplasm side of hexameric ring, around the central pore; | |
| Dibucaine | CVB3 | | A224V, I227V, A229V; | |  | | On a solvent accessible area on the cytoplasm side of hexameric ring, around the central pore; | |
| Zuclopenthixol | CVB3 | | A224V, I227V, A229V; | |  | | On a solvent accessible area on the cytoplasm side of hexameric ring, around the central pore; | |
| Pirlindole | CVB3 | | A224V, I227V, A229V; | |  | | On a solvent accessible area on the cytoplasm side of hexameric ring, around the central pore; | |

**References**

1. Pincus SE, Diamond DC, Emini EA, Wimmer E. Guanidine-selected mutants of poliovirus: mapping of point mutations to polypeptide 2C. J Virol. 1986;57(2):638-46. Epub 1986/02/01. PubMed PMID: 3003395; PubMed Central PMCID: PMCPmc252779.

2. Tolskaya EA, Romanova LI, Kolesnikova MS, Gmyl AP, Gorbalenya AE, Agol VI. Genetic studies on the poliovirus 2C protein, an NTPase. A plausible mechanism of guanidine effect on the 2C function and evidence for the importance of 2C oligomerization. Journal of molecular biology. 1994;236(5):1310-23. Epub 1994/03/11. PubMed PMID: 8126722.

3. Shimizu H, Agoh M, Agoh Y, Yoshida H, Yoshii K, Yoneyama T, et al. Mutations in the 2C region of poliovirus responsible for altered sensitivity to benzimidazole derivatives. J Virol. 2000;74(9):4146-54. Epub 2001/02/07. PubMed PMID: 10756027; PubMed Central PMCID: PMCPmc111929.

4. Klein M, Hadaschik D, Zimmermann H, Eggers HJ, Nelsen-Salz B. The picornavirus replication inhibitors HBB and guanidine in the echovirus-9 system: the significance of viral protein 2C. The Journal of general virology. 2000;81(Pt 4):895-901. Epub 2000/03/22. doi: 10.1099/0022-1317-81-4-895. PubMed PMID: 10725414.

5. De Palma AM, Heggermont W, Lanke K, Coutard B, Bergmann M, Monforte AM, et al. The thiazolobenzimidazole TBZE-029 inhibits enterovirus replication by targeting a short region immediately downstream from motif C in the nonstructural protein 2C. J Virol. 2008;82(10):4720-30. Epub 2008/03/14. doi: 10.1128/jvi.01338-07. PubMed PMID: 18337578; PubMed Central PMCID: PMCPmc2346740.

6. Vance LM, Moscufo N, Chow M, Heinz BA. Poliovirus 2C region functions during encapsidation of viral RNA. J Virol. 1997;71(11):8759-65. Epub 1997/10/29. PubMed PMID: 9343235; PubMed Central PMCID: PMCPmc192341.

7. Wang C, Jiang P, Sand C, Paul AV, Wimmer E. Alanine scanning of poliovirus 2CATPase reveals new genetic evidence that capsid protein/2CATPase interactions are essential for morphogenesis. J Virol. 2012;86(18):9964-75. Epub 2012/07/05. doi: 10.1128/jvi.00914-12. PubMed PMID: 22761387; PubMed Central PMCID: PMCPmc3446611.

8. Crotty S, Saleh MC, Gitlin L, Beske O, Andino R. The Poliovirus Replication Machinery Can Escape Inhibition by an Antiviral Drug That Targets a Host Cell Protein. Journal of Virology. 2004;78(7):3378-86. doi: 10.1128/jvi.78.7.3378-3386.2004.

9. Ulferts R, van der Linden L, Thibaut HJ, Lanke KH, Leyssen P, Coutard B, et al. Selective serotonin reuptake inhibitor fluoxetine inhibits replication of human enteroviruses B and D by targeting viral protein 2C. Antimicrob Agents Chemother. 2013;57(4):1952-6. Epub 2013/01/22. doi: 10.1128/aac.02084-12. PubMed PMID: 23335743; PubMed Central PMCID: PMCPmc3623316.

10. Ulferts R, de Boer SM, van der Linden L, Bauer L, Lyoo HR, Maté MJ, et al. Screening of a Library of FDA-Approved Drugs Identifies Several Enterovirus Replication Inhibitors That Target Viral Protein 2C. Antimicrobial Agents and Chemotherapy. 2016;60(5):2627-38. doi: 10.1128/aac.02182-15.
