# Supplementary material for: Crystal structure of a soluble fragment of poliovirus 2CATPase
Source: PLoS Pathog. 2018 Sep 19;14(9):e1007304. doi: 10.1371/journal.ppat.1007304 (PMC6166989; doi:10.1371/journal.ppat.1007304)
Supplement: S1 File — (PDF) [file ppat.1007304.s006.pdf]

Preliminary Full wwPDB X-ray Structure Validation Report (i)

Feb 23, 2018 – 06:45 AM EST

This is a Preliminary Full wwPDB X-ray Structure Validation Report.

This report is produced by the standalone wwPDB validation server.
The structure in question has not been deposited to the wwPDB.
This report should not be submitted to journals.

We welcome your comments at *validation@mail.wwpdb.org*
A user guide is available at
http://wwpdb.org/validation/2016/XrayValidationReportHelp
with specific help available everywhere you see the (i) symbol.

PRELIMINARY VALIDATION REPORT

The following versions of software and data (see references (i)) were used in the production of this report:

MolProbity	:	4.02b-467
Mogul	:	1.7.2 (RC1), CSD as538be (2017)
Xtriage (Phenix)	:	1.9-1692
EDS	:	rb-20030736
Percentile statistics	:	20161228.v01 (using entries in the PDB archive December 28th 2016)
Refmac	:	5.8.0135
CCP4	:	6.5.0
Ideal geometry (proteins)	:	Engh & Huber (2001)
Ideal geometry (DNA, RNA)	:	Parkinson et al. (1996)
Validation Pipeline (wwPDB-VP)	:	rb-20030736

1 Overall quality at a glance (i)

The following experimental techniques were used to determine the structure:
X-RAY DIFFRACTION

The reported resolution of this entry is 2.54 Å.

Percentile scores (ranging between 0-100) for global validation metrics of the entry are shown in the following graphic. The table shows the number of entries on which the scores are based.

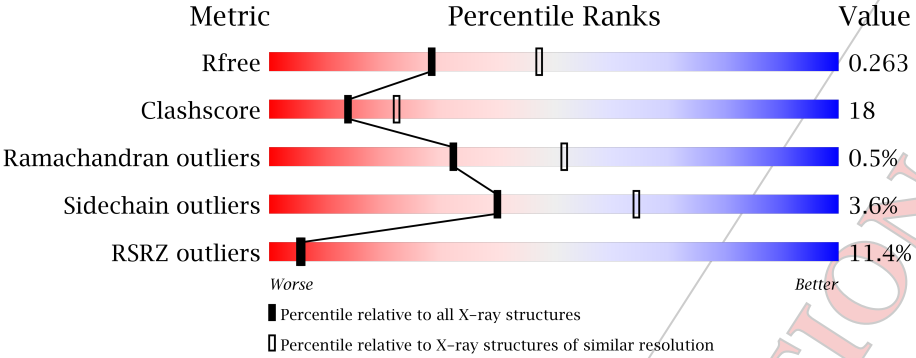

Metric	Whole archive (#Entries)	Similar resolution (#Entries, resolution range(Å))
R_{free}	100719	4993 (2.58-2.50)
Clashscore	112137	5755 (2.58-2.50)
Ramachandran outliers	110173	5652 (2.58-2.50)
Sidechain outliers	110143	5654 (2.58-2.50)
RSRZ outliers	101464	5026 (2.58-2.50)

The table below summarises the geometric issues observed across the polymeric chains and their fit to the electron density. The red, orange, yellow and green segments on the lower bar indicate the fraction of residues that contain outliers for >=3, 2, 1 and 0 types of geometric quality criteria. A grey segment represents the fraction of residues that are not modelled. The numeric value for each fraction is indicated below the corresponding segment, with a dot representing fractions <=5% The upper red bar (where present) indicates the fraction of residues that have poor fit to the electron density. The numeric value is given above the bar.

Mol	Chain	Length	Quality of chain
1	B	204	76% 23% •
2	A	202	.% 75% 23% •
3	D	202	3% 64% 35% •
4	C	188	15% 60% 37% ••
5	E	201	11% 56% 41% •

Continued on next page...

PRELIMINARY VALIDATION REPORT

Continued from previous page...

Mol	Chain	Length	Quality of chain
6	H	89	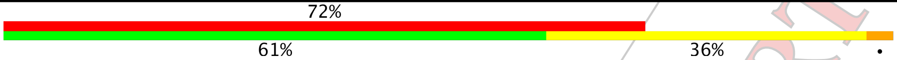

The following table lists non-polymeric compounds, carbohydrate monomers and non-standard residues in protein, DNA, RNA chains that are outliers for geometric or electron-density-fit criteria:

Mol	Type	Chain	Res	Chirality	Geometry	Clashes	Electron density
8	PO4	G	2	-	-	X	-

PRELIMINARY VALIDATION REPORT

2 Entry composition (i)

There are 9 unique types of molecules in this entry. The entry contains 16785 atoms, of which 8193 are hydrogens and 0 are deuteriums.

In the tables below, the ZeroOcc column contains the number of atoms modelled with zero occupancy, the AltConf column contains the number of residues with at least one atom in alternate conformation and the Trace column contains the number of residues modelled with at most 2 atoms.

- Molecule 1 is a protein.

Mol	Chain	Residues	Atoms						ZeroOcc	AltConf	Trace
			Total	C	H	N	O	S			
1	B	204	3110	974	1536	280	300	20	0	0	0

- Molecule 2 is a protein.

Mol	Chain	Residues	Atoms						ZeroOcc	AltConf	Trace
			Total	C	H	N	O	S			
2	A	202	3072	965	1515	275	297	20	0	0	0

- Molecule 3 is a protein.

Mol	Chain	Residues	Atoms						ZeroOcc	AltConf	Trace
			Total	C	H	N	O	S			
3	D	202	3074	963	1520	274	297	20	0	0	0

- Molecule 4 is a protein.

Mol	Chain	Residues	Atoms						ZeroOcc	AltConf	Trace
			Total	C	H	N	O	S			
4	C	188	2866	897	1419	257	273	20	0	0	0

- Molecule 5 is a protein.

Mol	Chain	Residues	Atoms						ZeroOcc	AltConf	Trace
			Total	C	H	N	O	S			
5	E	201	3062	958	1516	273	295	20	0	0	0

- Molecule 6 is a protein.

Mol	Chain	Residues	Atoms						ZeroOcc	AltConf	Trace
			Total	C	H	N	O	S			
6	H	89	1362	414	687	120	129	12	0	0	0

PRELIMINARY VALIDATION REPORT

- Molecule 7 is ZINC ION (three-letter code: ZN) (formula: Zn).

Mol	Chain	Residues	Atoms	ZeroOcc	AltConf
7	F	1	Total Zn<br>1 1	0	0
7	F	1	Total Zn<br>1 1	0	0
7	F	1	Total Zn<br>1 1	0	0
7	F	1	Total Zn<br>1 1	0	0
7	F	1	Total Zn<br>1 1	0	0
7	F	1	Total Zn<br>1 1	0	0

- Molecule 8 is PHOSPHATE ION (three-letter code: PO4) (formula: O_4P).

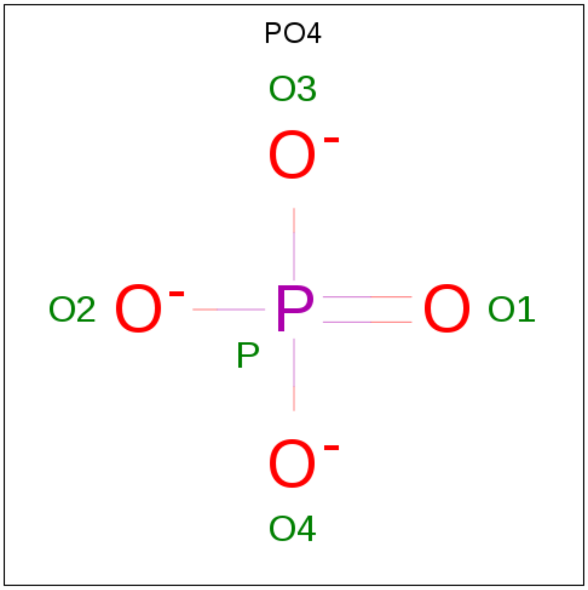

Mol	Chain	Residues	Atoms	ZeroOcc	AltConf
8	G	1	Total O P<br>5 4 1	0	0
8	G	1	Total O P<br>5 4 1	0	0
8	G	1	Total O P<br>5 4 1	0	0

- Molecule 9 is water.

PRELIMINARY VALIDATION REPORT

Mol	Chain	Residues	Atoms		ZeroOcc	AltConf
			Total	O		
9	S	218	218	218	0	0

PRELIMINARY VALIDATION REPORT

3 Residue-property plots (i)

These plots are drawn for all protein, RNA and DNA chains in the entry. The first graphic for a chain summarises the proportions of the various outlier classes displayed in the second graphic. The second graphic shows the sequence view annotated by issues in geometry and electron density. Residues are color-coded according to the number of geometric quality criteria for which they contain at least one outlier: green = 0, yellow = 1, orange = 2 and red = 3 or more. A red dot above a residue indicates a poor fit to the electron density (RSRZ > 2). Stretches of 2 or more consecutive residues without any outlier are shown as a green connector. Residues present in the sample, but not in the model, are shown in grey.

- Molecule 1:

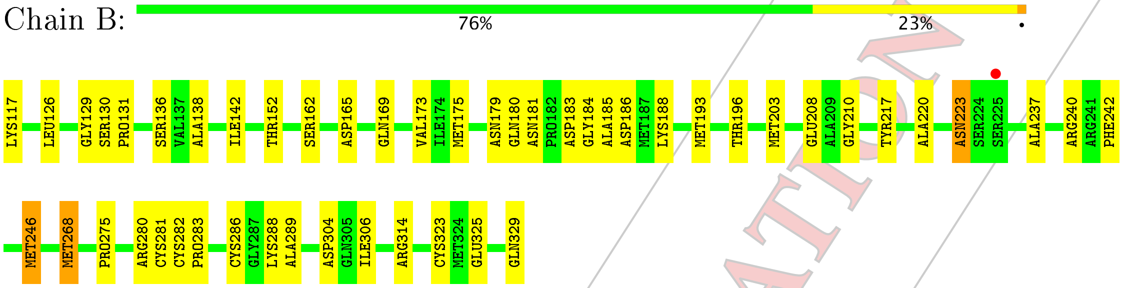

- Molecule 2:

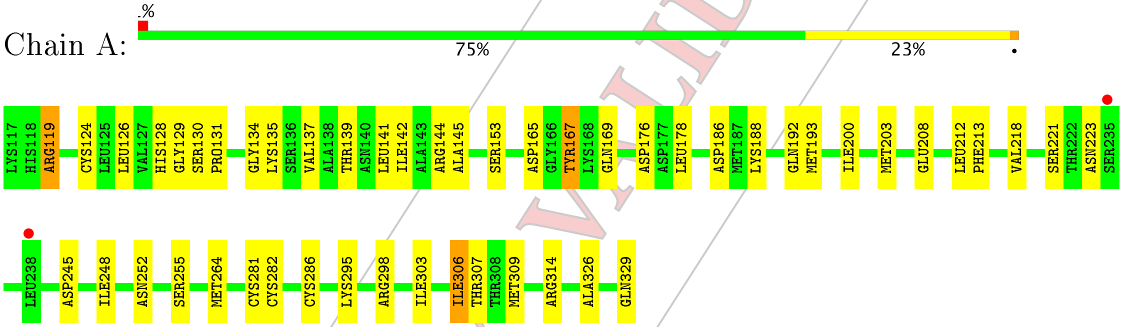

- Molecule 3:

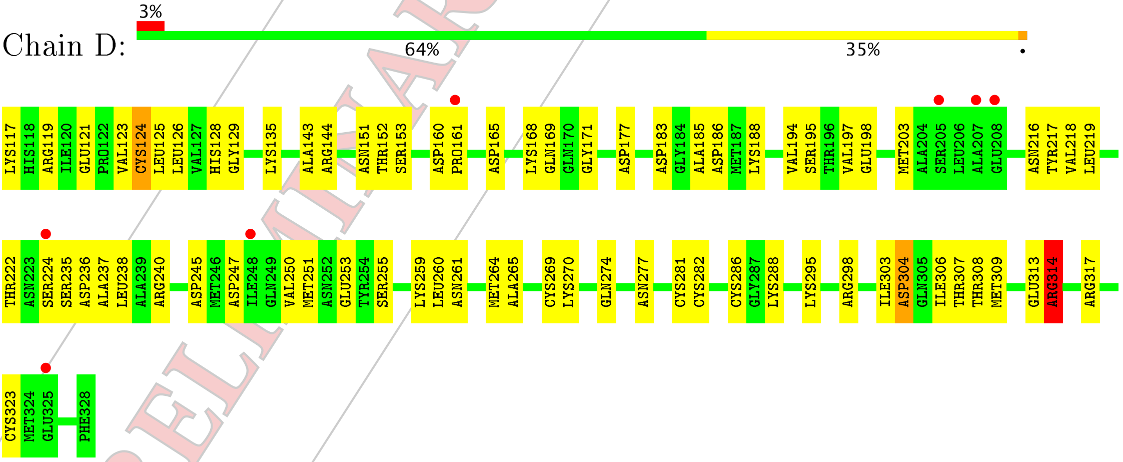

- Molecule 4:

PRELIMINARY VALIDATION REPORT

Chain C:

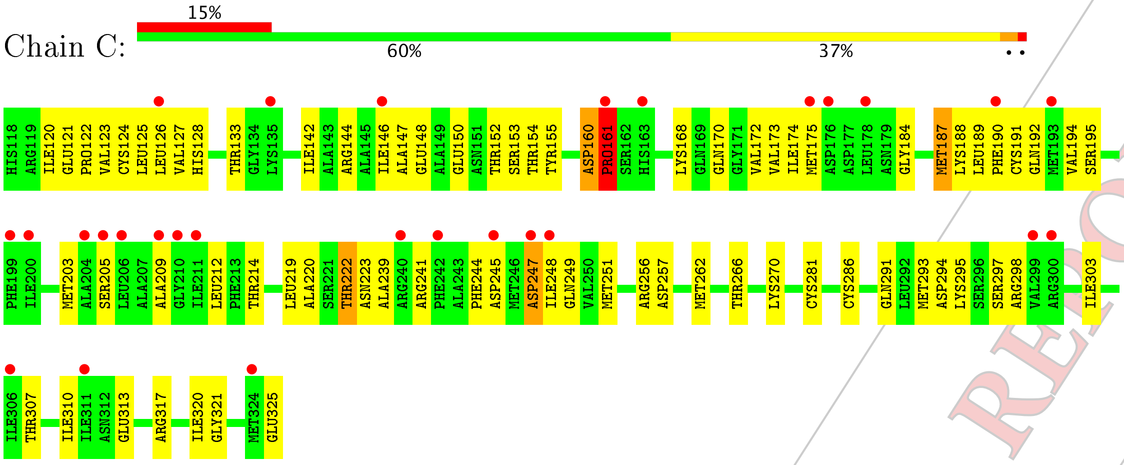

- Molecule 5:

Chain E:

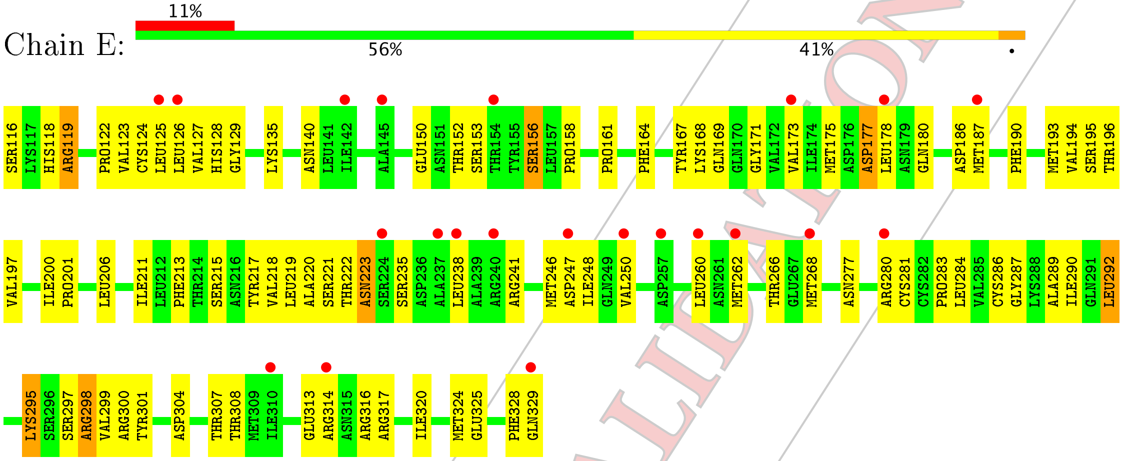

- Molecule 6:

Chain H:

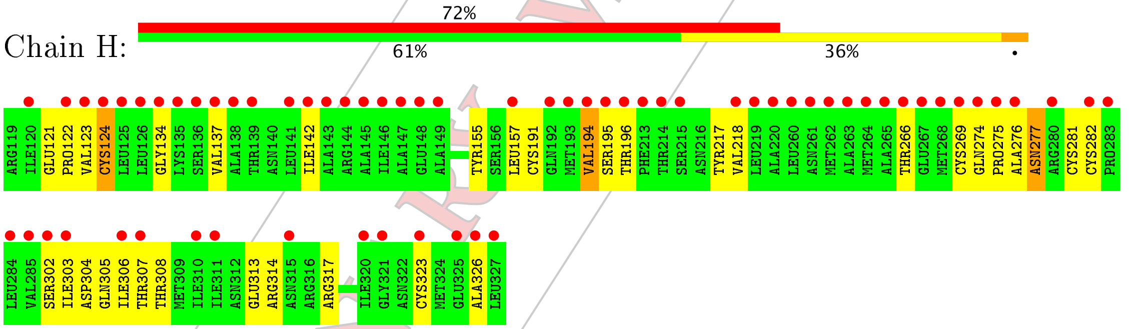

PRELIMINARY VALIDATION REPORT

4 Data and refinement statistics (i)

Property	Value	Source
Space group	P 1 21 1	Depositor
Cell constants<br>a, b, c, α, β, γ	52.70Å 84.07Å 172.56Å<br>90.00° 94.21° 90.00°	Depositor
Resolution (Å)	40.62 – 2.54<br>43.81 – 2.55	Depositor<br>EDS
% Data completeness<br>(in resolution range)	99.4 (40.62-2.54)<br>99.5 (43.81-2.55)	Depositor<br>EDS
R_{merge}	(Not available)	Depositor
R_{sym}	(Not available)	Depositor
$< I/\sigma(I) >$ [1]	1.23 (at 2.54Å)	Xtriage
Refinement program	PHENIX (1.10.1_2155: ???)	Depositor
R, R_{free}	0.202 , 0.265<br>0.197 , 0.263	Depositor<br>DCC
R_{free} test set	2521 reflections (5.12%)	DCC
Wilson B-factor (Å^2)	57.3	Xtriage
Anisotropy	0.153	Xtriage
Bulk solvent k_{sol}(e/Å^3), B_{sol}(Å^2)	0.37 , 60.6	EDS
L-test for twinning[2]	$< \|L\| >$ = 0.48, $< L^2 >$ = 0.31	Xtriage
Estimated twinning fraction	No twinning to report.	Xtriage
F_o,F_c correlation	0.93	EDS
Total number of atoms	16785	wwPDB-VP
Average B, all atoms (Å^2)	92.0	wwPDB-VP

Xtriage's analysis on translational NCS is as follows: *The largest off-origin peak in the Patterson function is 3.98% of the height of the origin peak. No significant pseudotranslation is detected.*

[1] Intensities estimated from amplitudes.
[2] Theoretical values of $< |L| >$, $< L^2 >$ for acentric reflections are 0.5, 0.333 respectively for untwinned datasets, and 0.375, 0.2 for perfectly twinned datasets.

PRELIMINARY VALIDATION REPORT

5 Model quality (i)

5.1 Standard geometry (i)

Bond lengths and bond angles in the following residue types are not validated in this section: ZN, PO4

The Z score for a bond length (or angle) is the number of standard deviations the observed value is removed from the expected value. A bond length (or angle) with $|Z| > 5$ is considered an outlier worth inspection. RMSZ is the root-mean-square of all Z scores of the bond lengths (or angles).

Mol	Chain	Bond lengths		Bond angles	
		RMSZ	$\#\|Z\| >5$	RMSZ	$\#\|Z\| >5$
1	B	0.94	1/1599 (0.1%)	0.87	2/2156 (0.1%)
2	A	0.93	2/1582 (0.1%)	0.98	5/2134 (0.2%)
3	D	0.91	1/1579 (0.1%)	0.88	4/2130 (0.2%)
4	C	0.68	0/1468	0.77	0/1976
5	E	0.69	1/1569 (0.1%)	0.78	1/2113 (0.0%)
6	H	0.47	0/672	0.59	0/896
All	All	0.82	5/8469 (0.1%)	0.84	12/11405 (0.1%)

All (5) bond length outliers are listed below:

Mol	Chain	Res	Type	Atoms	Z	Observed(Å)	Ideal(Å)
5	E	124	CYS	CB-SG	-6.63	1.71	1.82
2	A	282	CYS	CB-SG	-5.99	1.72	1.81
3	D	281	CYS	CB-SG	-5.99	1.72	1.81
1	B	217	TYR	CD1-CE1	-5.20	1.31	1.39
2	A	167	TYR	CE2-CZ	-5.11	1.31	1.38

All (12) bond angle outliers are listed below:

Mol	Chain	Res	Type	Atoms	Z	Observed(^{o})	Ideal(^{o})
2	A	119	ARG	NE-CZ-NH2	-12.11	114.24	120.30
2	A	119	ARG	NE-CZ-NH1	9.81	125.20	120.30
1	B	304	ASP	CB-CG-OD1	7.14	124.72	118.30
3	D	304	ASP	CB-CG-OD1	6.43	124.08	118.30
3	D	314	ARG	NE-CZ-NH1	-6.32	117.14	120.30
1	B	268	MET	CG-SD-CE	-5.61	91.23	100.20
3	D	124	CYS	CA-CB-SG	-5.54	104.02	114.00
2	A	306	ILE	CG1-CB-CG2	-5.31	99.72	111.40
2	A	245	ASP	CB-CG-OD2	-5.12	113.70	118.30
2	A	245	ASP	CB-CG-OD1	5.09	122.88	118.30

Continued on next page...

PRELIMINARY VALIDATION REPORT

Continued from previous page...

Mol	Chain	Res	Type	Atoms	Z	Observed(°)	Ideal(°)
5	E	119	ARG	NE-CZ-NH2	-5.09	117.76	120.30
3	D	304	ASP	CB-CG-OD2	-5.03	113.78	118.30

There are no chirality outliers.

There are no planarity outliers.

5.2 Too-close contacts (i)

In the following table, the Non-H and H(model) columns list the number of non-hydrogen atoms and hydrogen atoms in the chain respectively. The H(added) column lists the number of hydrogen atoms added and optimized by MolProbity. The Clashes column lists the number of clashes within the asymmetric unit, whereas Symm-Clashes lists symmetry related clashes.

Mol	Chain	Non-H	H(model)	H(added)	Clashes	Symm-Clashes
1	B	1574	1536	1554	33	3
2	A	1557	1515	1536	37	2
3	D	1554	1520	1533	61	0
4	C	1447	1419	1432	69	1
5	E	1546	1516	1528	80	0
6	H	675	687	681	25	0
7	F	6	0	0	0	0
8	G	15	0	0	2	0
9	S	218	0	0	60	0
All	All	8592	8193	8264	295	3

The all-atom clashscore is defined as the number of clashes found per 1000 atoms (including hydrogen atoms). The all-atom clashscore for this structure is 18.

All (295) close contacts within the same asymmetric unit are listed below, sorted by their clash magnitude.

Atom-1	Atom-2	Interatomic distance (Å)	Clash overlap (Å)
4:C:239:ALA:N	9:S:149:HOH:O	1.94	1.01
3:D:117:LYS:N	9:S:43:HOH:O	1.96	0.96
2:A:223:ASN:O	9:S:68:HOH:O	1.85	0.95
3:D:240:ARG:NH2	9:S:192:HOH:O	2.05	0.89
4:C:187:MET:SD	9:S:87:HOH:O	2.31	0.88
3:D:124:CYS:HA	9:S:159:HOH:O	1.76	0.86
4:C:128:HIS:ND1	9:S:140:HOH:O	2.10	0.84
4:C:325:GLU:O	9:S:130:HOH:O	1.95	0.83
3:D:298:ARG:NH2	9:S:174:HOH:O	2.14	0.79

Continued on next page...

PRELIMINARY VALIDATION REPORT

Continued from previous page...

Atom-1	Atom-2	Interatomic distance (Å)	Clash overlap (Å)
6:H:281:CYS:SG	6:H:282:CYS:N	2.55	0.79
5:E:197:VAL:O	9:S:62:HOH:O	2.01	0.79
2:A:141:LEU:O	9:S:111:HOH:O	2.01	0.78
4:C:244:PHE:O	9:S:81:HOH:O	2.02	0.78
6:H:155:TYR:N	9:S:91:HOH:O	2.19	0.75
3:D:123:VAL:O	9:S:159:HOH:O	2.03	0.75
4:C:126:LEU:O	9:S:81:HOH:O	2.04	0.74
3:D:152:THR:HG21	3:D:171:GLY:H	1.52	0.74
4:C:325:GLU:N	9:S:122:HOH:O	2.18	0.74
1:B:117:LYS:HB3	5:E:116:SER:N	2.03	0.73
2:A:203:MET:HE2	2:A:208:GLU:HB3	1.69	0.73
4:C:191:CYS:SG	4:C:241:ARG:NE	2.62	0.72
1:B:210:GLY:O	3:D:152:THR:HG23	1.89	0.72
2:A:144:ARG:N	9:S:111:HOH:O	2.22	0.71
1:B:165:ASP:HA	1:B:203:MET:HE3	1.73	0.71
3:D:119:ARG:NH1	3:D:197:VAL:O	2.24	0.70
3:D:152:THR:HG22	3:D:153:SER:H	1.55	0.70
3:D:270:LYS:NZ	9:S:188:HOH:O	2.24	0.70
5:E:223:ASN:ND2	5:E:223:ASN:O	2.24	0.70
5:E:250:VAL:N	9:S:214:HOH:O	2.18	0.70
2:A:134:GLY:N	8:G:2:PO4:O3	2.25	0.70
1:B:183:ASP:OD1	1:B:184:GLY:N	2.25	0.70
4:C:147:ALA:O	9:S:136:HOH:O	2.10	0.70
3:D:218:VAL:O	9:S:159:HOH:O	2.09	0.70
1:B:208:GLU:OE1	9:S:46:HOH:O	2.10	0.69
3:D:303:ILE:O	3:D:307:THR:HG22	1.93	0.69
6:H:191:CYS:SG	9:S:165:HOH:O	2.51	0.67
5:E:119:ARG:NH2	5:E:193:MET:O	2.27	0.67
4:C:144:ARG:NH2	9:S:108:HOH:O	2.27	0.67
4:C:191:CYS:O	4:C:195:SER:OG	2.13	0.67
3:D:237:ALA:N	9:S:48:HOH:O	2.28	0.66
4:C:245:ASP:CG	4:C:295:LYS:HE2	2.16	0.66
1:B:165:ASP:HA	1:B:203:MET:CE	2.26	0.66
2:A:326:ALA:O	9:S:104:HOH:O	2.13	0.65
4:C:294:ASP:HB3	4:C:297:SER:HB2	1.78	0.65
1:B:169:GLN:OE1	9:S:12:HOH:O	2.15	0.64
5:E:156:SER:OG	9:S:47:HOH:O	2.15	0.64
1:B:281:CYS:SG	1:B:286:CYS:HB3	2.38	0.64
3:D:135:LYS:NZ	3:D:222:THR:O	2.27	0.64
2:A:303:ILE:O	2:A:307:THR:HG23	1.98	0.63
4:C:222:THR:HG23	9:S:140:HOH:O	1.99	0.62

Continued on next page...

PRELIMINARY VALIDATION REPORT

Continued from previous page...

Atom-1	Atom-2	Interatomic distance (Å)	Clash overlap (Å)
1:B:325:GLU:O	1:B:329:GLN:HB2	1.99	0.62
2:A:142:ILE:C	9:S:111:HOH:O	2.38	0.61
1:B:203:MET:HE2	1:B:208:GLU:HB3	1.82	0.61
2:A:281:CYS:SG	2:A:286:CYS:HB3	2.41	0.61
6:H:121:GLU:OE2	6:H:317:ARG:NH1	2.33	0.61
4:C:223:ASN:N	9:S:140:HOH:O	2.33	0.61
5:E:219:LEU:N	5:E:219:LEU:HD12	2.15	0.60
5:E:177:ASP:OD1	5:E:177:ASP:N	2.33	0.60
4:C:256:ARG:O	4:C:257:ASP:HB3	2.01	0.60
5:E:140:ASN:ND2	9:S:100:HOH:O	2.34	0.60
1:B:268:MET:HE1	9:S:104:HOH:O	2.02	0.60
1:B:117:LYS:HB3	5:E:116:SER:CA	2.33	0.59
4:C:245:ASP:OD2	4:C:295:LYS:HE2	2.02	0.59
3:D:245:ASP:OD2	3:D:295:LYS:HD3	2.03	0.58
3:D:277:ASN:OD1	3:D:308:THR:HG22	2.04	0.58
4:C:146:ILE:HG13	4:C:172:VAL:HG11	1.86	0.58
5:E:180:GLN:HA	5:E:187:MET:HG3	1.86	0.58
3:D:217:TYR:CZ	3:D:314:ARG:HD2	2.38	0.58
5:E:119:ARG:NE	9:S:62:HOH:O	2.27	0.57
3:D:151:ASN:ND2	9:S:127:HOH:O	2.36	0.57
5:E:167:TYR:CZ	5:E:169:GLN:HA	2.39	0.57
5:E:200:ILE:O	5:E:200:ILE:HG23	2.03	0.57
5:E:297:SER:OG	5:E:299:VAL:HG12	2.04	0.57
4:C:152:THR:C	9:S:136:HOH:O	2.42	0.57
3:D:261:ASN:ND2	3:D:264:MET:SD	2.78	0.57
6:H:276:ALA:N	6:H:304:ASP:OD2	2.34	0.57
1:B:280:ARG:NE	9:S:104:HOH:O	2.37	0.57
4:C:147:ALA:C	9:S:136:HOH:O	2.43	0.56
4:C:121:GLU:O	4:C:317:ARG:NH1	2.37	0.56
3:D:314:ARG:NH1	9:S:53:HOH:O	2.38	0.56
1:B:138:ALA:O	1:B:142:ILE:HG13	2.05	0.56
1:B:185:ALA:HA	1:B:188:LYS:HE3	1.87	0.56
4:C:142:ILE:HG23	4:C:307:THR:HG22	1.87	0.56
3:D:216:ASN:HB3	3:D:314:ARG:HE	1.69	0.56
5:E:152:THR:HG22	5:E:153:SER:H	1.71	0.56
4:C:245:ASP:HA	9:S:81:HOH:O	2.04	0.56
5:E:152:THR:HG21	5:E:171:GLY:H	1.69	0.56
4:C:189:LEU:HA	4:C:192:GLN:OE1	2.06	0.55
3:D:304:ASP:OD2	9:S:37:HOH:O	2.18	0.55
2:A:203:MET:CE	2:A:208:GLU:HB3	2.37	0.55
4:C:297:SER:O	4:C:298:ARG:HB3	2.05	0.55

Continued on next page...

PRELIMINARY VALIDATION REPORT

Continued from previous page...

Atom-1	Atom-2	Interatomic distance (Å)	Clash overlap (Å)
5:E:156:SER:N	9:S:47:HOH:O	2.39	0.55
2:A:203:MET:HE2	2:A:208:GLU:CB	2.35	0.55
5:E:248:ILE:HG13	5:E:290:ILE:HD11	1.89	0.55
5:E:125:LEU:HD21	5:E:127:VAL:HG13	1.88	0.55
1:B:179:ASN:N	1:B:186:ASP:OD2	2.39	0.54
3:D:185:ALA:O	3:D:188:LYS:N	2.39	0.54
5:E:284:LEU:HA	5:E:290:ILE:HG22	1.89	0.54
4:C:150:GLU:OE2	9:S:134:HOH:O	2.18	0.54
4:C:293:MET:HE1	4:C:298:ARG:NH1	2.23	0.54
4:C:170:GLN:N	4:C:170:GLN:OE1	2.37	0.53
4:C:281:CYS:SG	4:C:286:CYS:HB3	2.47	0.53
3:D:168:LYS:O	3:D:169:GLN:HB2	2.08	0.53
5:E:213:PHE:CZ	5:E:215:SER:HB2	2.43	0.53
1:B:286:CYS:SG	1:B:288:LYS:HG3	2.49	0.53
6:H:269:CYS:SG	6:H:281:CYS:SG	3.04	0.53
3:D:247:ASP:OD2	3:D:295:LYS:HG2	2.09	0.52
2:A:326:ALA:C	9:S:104:HOH:O	2.46	0.52
5:E:219:LEU:N	5:E:219:LEU:CD1	2.72	0.52
3:D:277:ASN:OD1	3:D:307:THR:HG23	2.09	0.52
4:C:293:MET:HE3	4:C:298:ARG:CZ	2.39	0.52
4:C:120:ILE:HD11	4:C:317:ARG:HB3	1.92	0.52
2:A:124:CYS:HA	2:A:218:VAL:O	2.10	0.52
3:D:250:VAL:HG12	3:D:251:MET:O	2.10	0.51
5:E:135:LYS:HG2	5:E:221:SER:HB2	1.92	0.51
1:B:246:MET:HE3	1:B:306:ILE:HD11	1.92	0.51
4:C:249:GLN:NE2	4:C:293:MET:SD	2.83	0.51
4:C:155:TYR:O	4:C:173:VAL:HG23	2.10	0.51
2:A:188:LYS:O	2:A:192:GLN:HG3	2.11	0.51
2:A:200:ILE:HG12	2:A:212:LEU:HD23	1.93	0.51
3:D:128:HIS:ND1	3:D:224:SER:HB2	2.26	0.50
6:H:134:GLY:N	9:S:117:HOH:O	2.44	0.50
6:H:191:CYS:N	9:S:66:HOH:O	2.43	0.50
5:E:127:VAL:HG23	5:E:135:LYS:HG3	1.92	0.50
3:D:235:SER:O	3:D:238:LEU:HG	2.11	0.50
5:E:316:ARG:O	5:E:320:ILE:HG12	2.12	0.50
3:D:217:TYR:CE1	3:D:314:ARG:HD2	2.47	0.50
5:E:287:GLY:HA2	5:E:290:ILE:O	2.12	0.50
4:C:303:ILE:O	4:C:307:THR:HG23	2.10	0.50
5:E:158:PRO:O	5:E:161:PRO:HB3	2.11	0.50
4:C:191:CYS:SG	4:C:241:ARG:CZ	2.99	0.50
2:A:142:ILE:HG23	2:A:307:THR:HG22	1.94	0.49

Continued on next page...

PRELIMINARY VALIDATION REPORT

Continued from previous page...

Atom-1	Atom-2	Interatomic distance (Å)	Clash overlap (Å)
5:E:248:ILE:HD11	5:E:290:ILE:CG1	2.42	0.49
6:H:269:CYS:HG	6:H:281:CYS:HG	1.52	0.49
3:D:218:VAL:N	9:S:159:HOH:O	2.03	0.49
2:A:165:ASP:HA	2:A:203:MET:CE	2.42	0.49
1:B:117:LYS:O	1:B:117:LYS:HG2	2.13	0.49
1:B:283:PRO:HB2	1:B:289:ALA:HB2	1.93	0.49
4:C:125:LEU:HD13	4:C:310:ILE:HD11	1.93	0.49
3:D:306:ILE:HD12	3:D:309:MET:CE	2.42	0.49
6:H:277:ASN:OD1	6:H:304:ASP:OD1	2.30	0.49
2:A:306:ILE:HA	2:A:309:MET:HE3	1.95	0.49
6:H:274:GLN:OE1	6:H:275:PRO:HD2	2.12	0.49
5:E:218:VAL:C	5:E:219:LEU:HD12	2.33	0.49
5:E:246:MET:HG3	5:E:292:LEU:HD13	1.95	0.49
3:D:124:CYS:SG	3:D:125:LEU:N	2.85	0.49
4:C:223:ASN:C	9:S:140:HOH:O	2.51	0.48
6:H:277:ASN:OD1	6:H:307:THR:OG1	2.32	0.48
4:C:133:THR:HB	4:C:248:ILE:HD13	1.96	0.48
2:A:135:LYS:O	2:A:139:THR:HG23	2.13	0.48
4:C:175:MET:HB3	4:C:220:ALA:HB2	1.96	0.48
3:D:253:GLU:H	3:D:253:GLU:CD	2.16	0.48
5:E:116:SER:HB2	5:E:118:HIS:CE1	2.49	0.48
4:C:184:GLY:O	4:C:188:LYS:HG2	2.13	0.48
3:D:165:ASP:HA	3:D:203:MET:CE	2.43	0.48
2:A:252:ASN:O	2:A:255:SER:OG	2.32	0.47
3:D:216:ASN:CB	3:D:314:ARG:HE	2.27	0.47
5:E:195:SER:OG	5:E:196:THR:N	2.47	0.47
1:B:126:LEU:HD12	1:B:242:PHE:CE1	2.49	0.47
5:E:200:ILE:HD12	5:E:211:ILE:O	2.14	0.47
2:A:223:ASN:N	2:A:223:ASN:OD1	2.48	0.47
3:D:165:ASP:HA	3:D:203:MET:HE3	1.96	0.47
5:E:325:GLU:HA	5:E:325:GLU:OE1	2.15	0.47
6:H:195:SER:OG	6:H:196:THR:N	2.47	0.47
4:C:212:LEU:HD13	5:E:206:LEU:HD21	1.97	0.47
2:A:264:MET:HE3	9:S:116:HOH:O	2.15	0.47
5:E:173:VAL:HG11	5:E:193:MET:CE	2.45	0.47
5:E:328:PHE:O	5:E:329:GLN:HB2	2.14	0.47
3:D:143:ALA:HB2	3:D:219:LEU:HD12	1.97	0.46
5:E:277:ASN:HD21	5:E:308:THR:HG22	1.80	0.46
1:B:237:ALA:O	1:B:240:ARG:HG2	2.15	0.46
4:C:293:MET:CE	4:C:298:ARG:CZ	2.93	0.46
6:H:303:ILE:O	6:H:306:ILE:HG22	2.14	0.46

Continued on next page...

PRELIMINARY VALIDATION REPORT

Continued from previous page...

Atom-1	Atom-2	Interatomic distance (Å)	Clash overlap (Å)
5:E:194:VAL:HG13	5:E:218:VAL:HG21	1.97	0.46
5:E:295:LYS:NZ	9:S:215:HOH:O	2.42	0.46
2:A:130:SER:HB2	2:A:131:PRO:HD2	1.97	0.46
4:C:123:VAL:HG22	4:C:313:GLU:HG3	1.97	0.46
4:C:128:HIS:CB	9:S:140:HOH:O	2.63	0.46
5:E:126:LEU:HA	5:E:220:ALA:O	2.15	0.46
5:E:152:THR:HG21	5:E:171:GLY:N	2.31	0.46
4:C:160:ASP:N	4:C:161:PRO:HD3	2.30	0.45
4:C:154:THR:HG22	4:C:172:VAL:CG2	2.46	0.45
3:D:247:ASP:OD2	3:D:295:LYS:CG	2.64	0.45
4:C:168:LYS:HB3	5:E:200:ILE:HG21	1.98	0.45
6:H:155:TYR:HE1	6:H:157:LEU:CD2	2.30	0.45
1:B:246:MET:SD	1:B:306:ILE:HD11	2.56	0.45
4:C:262:MET:HE3	4:C:266:THR:HG23	1.98	0.45
5:E:129:GLY:O	5:E:223:ASN:HB3	2.17	0.45
5:E:281:CYS:SG	5:E:286:CYS:HB3	2.55	0.45
6:H:217:TYR:CZ	6:H:314:ARG:HD2	2.50	0.45
2:A:134:GLY:O	2:A:137:VAL:HG12	2.17	0.45
4:C:128:HIS:ND1	4:C:222:THR:HG23	2.32	0.45
1:B:180:GLN:HG3	1:B:181:ASN:N	2.32	0.45
6:H:323:CYS:O	6:H:326:ALA:N	2.47	0.45
4:C:214:THR:HG22	4:C:214:THR:O	2.18	0.44
1:B:280:ARG:NH2	2:A:329:GLN:O	2.50	0.44
3:D:260:LEU:HD23	3:D:260:LEU:C	2.37	0.44
4:C:152:THR:N	9:S:136:HOH:O	2.27	0.44
4:C:175:MET:HE1	4:C:190:PHE:HD2	1.82	0.44
1:B:130:SER:HB3	1:B:131:PRO:HD2	1.99	0.44
3:D:277:ASN:OD1	3:D:308:THR:CG2	2.65	0.44
5:E:222:THR:HG22	5:E:223:ASN:N	2.33	0.44
5:E:283:PRO:HB2	5:E:289:ALA:HB2	2.00	0.44
2:A:128:HIS:O	2:A:248:ILE:HG22	2.18	0.44
1:B:117:LYS:HB3	5:E:116:SER:HB3	1.99	0.44
5:E:235:SER:HA	5:E:238:LEU:CD2	2.48	0.44
4:C:147:ALA:CA	9:S:136:HOH:O	2.66	0.44
3:D:123:VAL:HG22	3:D:313:GLU:HG3	1.99	0.44
2:A:298:ARG:NH2	9:S:112:HOH:O	2.36	0.44
4:C:174:ILE:HA	4:C:219:LEU:O	2.18	0.44
5:E:150:GLU:OE1	5:E:217:TYR:OH	2.28	0.44
5:E:313:GLU:O	5:E:317:ARG:HG3	2.18	0.44
6:H:123:VAL:HG12	6:H:124:CYS:N	2.33	0.44
3:D:197:VAL:O	3:D:198:GLU:C	2.56	0.43

Continued on next page...

PRELIMINARY VALIDATION REPORT

Continued from previous page...

Atom-1	Atom-2	Interatomic distance (Å)	Clash overlap (Å)
5:E:119:ARG:NH2	9:S:62:HOH:O	2.51	0.43
3:D:255:SER:HA	3:D:259:LYS:O	2.19	0.43
5:E:164:PHE:HB2	5:E:201:PRO:HB2	2.00	0.43
5:E:299:VAL:CG2	5:E:300:ARG:N	2.81	0.43
2:A:203:MET:CE	2:A:208:GLU:CB	2.95	0.43
1:B:173:VAL:HG11	1:B:193:MET:CE	2.48	0.43
3:D:129:GLY:O	3:D:135:LYS:HE2	2.17	0.43
1:B:129:GLY:O	1:B:223:ASN:OD1	2.36	0.43
5:E:277:ASN:OD1	5:E:308:THR:HG23	2.18	0.43
2:A:193:MET:HE2	2:A:213:PHE:CZ	2.54	0.43
5:E:299:VAL:HG22	5:E:300:ARG:N	2.33	0.43
4:C:123:VAL:HG11	4:C:310:ILE:HG23	2.01	0.43
3:D:121:GLU:O	3:D:317:ARG:NH1	2.52	0.43
3:D:152:THR:HG21	3:D:171:GLY:N	2.28	0.43
3:D:126:LEU:HD11	3:D:222:THR:HG22	2.00	0.43
5:E:268:MET:HE2	5:E:280:ARG:HD3	2.01	0.43
4:C:184:GLY:O	4:C:187:MET:HG3	2.19	0.43
5:E:173:VAL:HG11	5:E:193:MET:HE1	2.01	0.43
5:E:297:SER:O	5:E:298:ARG:CB	2.66	0.43
3:D:269:CYS:SG	3:D:282:CYS:HA	2.59	0.42
4:C:122:PRO:HG2	4:C:194:VAL:HA	2.02	0.42
5:E:168:LYS:HA	5:E:168:LYS:HD2	1.85	0.42
2:A:306:ILE:HD12	2:A:309:MET:HE3	2.00	0.42
1:B:175:MET:O	1:B:220:ALA:HA	2.19	0.42
3:D:218:VAL:CA	9:S:159:HOH:O	2.60	0.42
2:A:178:LEU:HD12	2:A:186:ASP:HB3	1.99	0.42
4:C:251:MET:SD	4:C:291:GLN:HG3	2.60	0.42
5:E:248:ILE:HD11	5:E:290:ILE:HG12	1.99	0.42
5:E:218:VAL:HG23	5:E:218:VAL:O	2.19	0.42
5:E:152:THR:CG2	5:E:153:SER:H	2.30	0.42
5:E:194:VAL:HG23	5:E:194:VAL:O	2.20	0.42
5:E:250:VAL:HG12	9:S:214:HOH:O	2.19	0.42
3:D:260:LEU:HD21	3:D:265:ALA:CB	2.49	0.42
3:D:306:ILE:HD12	3:D:309:MET:HE3	2.01	0.42
5:E:128:HIS:O	5:E:248:ILE:HG22	2.20	0.42
2:A:135:LYS:HB3	2:A:221:SER:HB2	2.02	0.42
3:D:121:GLU:OE1	3:D:194:VAL:HG12	2.19	0.42
4:C:127:VAL:HG13	4:C:248:ILE:CG2	2.50	0.42
5:E:194:VAL:O	5:E:241:ARG:NH1	2.53	0.42
6:H:217:TYR:O	6:H:218:VAL:HG23	2.19	0.42
5:E:122:PRO:HG2	5:E:194:VAL:HG12	2.01	0.41

Continued on next page...

PRELIMINARY VALIDATION REPORT

Continued from previous page...

Atom-1	Atom-2	Interatomic distance (Å)	Clash overlap (Å)
5:E:262:MET:O	5:E:266:THR:HG23	2.20	0.41
6:H:134:GLY:O	6:H:137:VAL:HB	2.20	0.41
6:H:313:GLU:OE1	6:H:313:GLU:O	2.37	0.41
6:H:305:GLN:O	6:H:308:THR:OG1	2.29	0.41
6:H:142:ILE:HG23	6:H:307:THR:HG22	2.02	0.41
5:E:190:PHE:C	5:E:190:PHE:CD1	2.94	0.41
2:A:126:LEU:HD22	2:A:128:HIS:NE2	2.36	0.41
2:A:145:ALA:N	9:S:111:HOH:O	2.05	0.41
4:C:128:HIS:CG	9:S:140:HOH:O	2.69	0.41
3:D:152:THR:CG2	3:D:171:GLY:H	2.27	0.41
5:E:299:VAL:HG13	5:E:301:TYR:CZ	2.56	0.41
5:E:175:MET:HB3	5:E:178:LEU:HD21	2.03	0.41
3:D:152:THR:HG22	3:D:153:SER:N	2.27	0.41
5:E:247:ASP:OD1	5:E:295:LYS:CG	2.69	0.41
1:B:282:CYS:HB2	1:B:283:PRO:HD2	2.03	0.41
2:A:167:TYR:CZ	2:A:169:GLN:HA	2.55	0.41
4:C:293:MET:CE	4:C:298:ARG:NH1	2.84	0.41
3:D:144:ARG:HD3	5:E:324:MET:SD	2.60	0.41
3:D:185:ALA:O	3:D:186:ASP:C	2.58	0.41
5:E:304:ASP:O	5:E:307:THR:OG1	2.39	0.41
4:C:152:THR:CA	9:S:136:HOH:O	2.69	0.41
4:C:320:ILE:CG1	4:C:321:GLY:N	2.84	0.41
3:D:160:ASP:N	3:D:161:PRO:HD3	2.36	0.41
5:E:260:LEU:HA	5:E:260:LEU:HD23	1.95	0.41
2:A:135:LYS:N	8:G:2:PO4:O3	2.53	0.41
3:D:129:GLY:HA3	3:D:135:LYS:HD3	2.03	0.41
4:C:203:MET:HG3	4:C:209:ALA:N	2.37	0.40
1:B:323:CYS:HB2	4:C:266:THR:HB	2.02	0.40
4:C:247:ASP:OD1	4:C:295:LYS:HG2	2.22	0.40
3:D:286:CYS:SG	3:D:288:LYS:HG2	2.62	0.40
5:E:178:LEU:HA	5:E:186:ASP:OD2	2.21	0.40
4:C:168:LYS:CB	5:E:200:ILE:HG21	2.51	0.40
6:H:122:PRO:HG2	6:H:194:VAL:HA	2.02	0.40
1:B:117:LYS:HG3	1:B:196:THR:CG2	2.51	0.40
4:C:126:LEU:HA	4:C:220:ALA:O	2.21	0.40
3:D:251:MET:HG3	3:D:288:LYS:O	2.22	0.40
5:E:123:VAL:CG2	5:E:217:TYR:CD2	3.05	0.40
2:A:129:GLY:O	2:A:223:ASN:CB	2.70	0.40
4:C:147:ALA:O	4:C:148:GLU:C	2.57	0.40
6:H:124:CYS:SG	6:H:194:VAL:HG21	2.61	0.40

All (3) symmetry-related close contacts are listed below. The label for Atom-2 includes the sym-

PRELIMINARY VALIDATION REPORT

metry operator and encoded unit-cell translations to be applied.

Atom-1	Atom-2	Interatomic distance (Å)	Clash overlap (Å)
1:B:183:ASP:OD2	4:C:270:LYS:HZ2[1_455]	1.38	0.22
1:B:275:PRO:O	2:A:298:ARG:HH21[1_655]	1.39	0.21
1:B:275:PRO:O	2:A:298:ARG:NH2[1_655]	2.01	0.19

5.3 Torsion angles (i)

5.3.1 Protein backbone (i)

In the following table, the Percentiles column shows the percent Ramachandran outliers of the chain as a percentile score with respect to all X-ray entries followed by that with respect to entries of similar resolution.

The Analysed column shows the number of residues for which the backbone conformation was analysed, and the total number of residues.

Mol	Chain	Analysed	Favoured	Allowed	Outliers	Percentiles	
1	B	200/204 (98%)	191 (96%)	9 (4%)	0	100	100
2	A	198/202 (98%)	191 (96%)	7 (4%)	0	100	100
3	D	198/202 (98%)	183 (92%)	14 (7%)	1 (0%)	32	52
4	C	180/188 (96%)	161 (89%)	17 (9%)	2 (1%)	17	28
5	E	195/201 (97%)	186 (95%)	8 (4%)	1 (0%)	32	52
6	H	71/89 (80%)	65 (92%)	5 (7%)	1 (1%)	13	22
All	All	1042/1086 (96%)	977 (94%)	60 (6%)	5 (0%)	32	52

All (5) Ramachandran outliers are listed below:

Mol	Chain	Res	Type
3	D	177	ASP
5	E	298	ARG
4	C	161	PRO
4	C	222	THR
6	H	194	VAL

5.3.2 Protein sidechains (i)

In the following table, the Percentiles column shows the percent sidechain outliers of the chain as a percentile score with respect to all X-ray entries followed by that with respect to entries of similar resolution.

PRELIMINARY VALIDATION REPORT

The Analysed column shows the number of residues for which the sidechain conformation was analysed, and the total number of residues.

Mol	Chain	Analysed	Rotameric	Outliers	Percentiles	
1	B	176/176 (100%)	170 (97%)	6 (3%)	42	67
2	A	174/174 (100%)	169 (97%)	5 (3%)	48	72
3	D	174/174 (100%)	168 (97%)	6 (3%)	42	67
4	C	162/162 (100%)	155 (96%)	7 (4%)	33	56
5	E	173/173 (100%)	167 (96%)	6 (4%)	41	65
6	H	77/77 (100%)	73 (95%)	4 (5%)	27	47
All	All	936/936 (100%)	902 (96%)	34 (4%)	40	64

All (34) residues with a non-rotameric sidechain are listed below:

Mol	Chain	Res	Type
1	B	136	SER
1	B	152	THR
1	B	162	SER
1	B	223	ASN
1	B	246	MET
1	B	314	ARG
2	A	119	ARG
2	A	153	SER
2	A	176	ASP
2	A	295	LYS
2	A	314	ARG
3	D	183	ASP
3	D	195	SER
3	D	236	ASP
3	D	274	GLN
3	D	314	ARG
3	D	323	CYS
4	C	124	CYS
4	C	153	SER
4	C	160	ASP
4	C	161	PRO
4	C	187	MET
4	C	205	SER
4	C	247	ASP
5	E	156	SER
5	E	177	ASP
5	E	223	ASN

Continued on next page...

PRELIMINARY VALIDATION REPORT

Continued from previous page...

Mol	Chain	Res	Type
5	E	292	LEU
5	E	295	LYS
5	E	314	ARG
6	H	124	CYS
6	H	266	THR
6	H	277	ASN
6	H	302	SER

Some sidechains can be flipped to improve hydrogen bonding and reduce clashes. All (2) such sidechains are listed below:

Mol	Chain	Res	Type
1	B	223	ASN
5	E	118	HIS

5.3.3 RNA (i)

There are no RNA molecules in this entry.

5.4 Non-standard residues in protein, DNA, RNA chains (i)

There are no non-standard protein/DNA/RNA residues in this entry.

5.5 Carbohydrates (i)

There are no carbohydrates in this entry.

5.6 Ligand geometry (i)

Of 9 ligands modelled in this entry, 6 are monoatomic - leaving 3 for Mogul analysis.

In the following table, the Counts columns list the number of bonds (or angles) for which Mogul statistics could be retrieved, the number of bonds (or angles) that are observed in the model and the number of bonds (or angles) that are defined in the Chemical Component Dictionary. The Link column lists molecule types, if any, to which the group is linked. The Z score for a bond length (or angle) is the number of standard deviations the observed value is removed from the expected value. A bond length (or angle) with $|Z| > 2$ is considered an outlier worth inspection. RMSZ is the root-mean-square of all Z scores of the bond lengths (or angles).

PRELIMINARY VALIDATION REPORT

Mol	Type	Chain	Res	Link	Bond lengths			Bond angles		
					Counts	RMSZ	$\#\|Z\|>2$	Counts	RMSZ	$\#\|Z\|>2$
8	PO4	G	1	-	4,4,4	0.76	0	6,6,6	0.39	0
8	PO4	G	2	-	4,4,4	0.77	0	6,6,6	0.39	0
8	PO4	G	3	-	4,4,4	0.75	0	6,6,6	0.39	0

In the following table, the Chirals column lists the number of chiral outliers, the number of chiral centers analysed, the number of these observed in the model and the number defined in the Chemical Component Dictionary. Similar counts are reported in the Torsion and Rings columns. '-' means no outliers of that kind were identified.

Mol	Type	Chain	Res	Link	Chirals	Torsions	Rings
8	PO4	G	1	-	-	0/0/0/0	0/0/0/0
8	PO4	G	2	-	-	0/0/0/0	0/0/0/0
8	PO4	G	3	-	-	0/0/0/0	0/0/0/0

There are no bond length outliers.

There are no bond angle outliers.

There are no chirality outliers.

There are no torsion outliers.

There are no ring outliers.

1 monomer is involved in 2 short contacts:

Mol	Chain	Res	Type	Clashes	Symm-Clashes
8	G	2	PO4	2	0

5.7 Other polymers (i)

There are no such residues in this entry.

5.8 Polymer linkage issues (i)

The following chains have linkage breaks:

Mol	Chain	Number of breaks
6	H	8
4	C	3
5	E	2
3	D	1
1	B	1
2	A	1

PRELIMINARY VALIDATION REPORT

All chain breaks are listed below:

Model	Chain	Residue-1	Atom-1	Residue-2	Atom-2	Distance (Å)
1	A	223:ASN	C	235:SER	N	18.77
1	H	220:ALA	C	260:LEU	N	16.80
1	D	224:SER	C	235:SER	N	16.72
1	C	223:ASN	C	239:ALA	N	15.19
1	E	224:SER	C	235:SER	N	15.05
1	H	157:LEU	C	191:CYS	N	12.80
1	H	126:LEU	C	134:GLY	N	12.33
1	H	151:ASN	C	155:TYR	N	10.71
1	H	196:THR	C	213:PHE	N	9.43
1	B	226:ARG	C	236:ASP	N	9.40
1	H	269:CYS	C	274:GLN	N	9.27
1	C	179:ASN	C	184:GLY	N	8.42
1	H	277:ASN	C	280:ARG	N	6.64
1	E	180:GLN	C	184:GLY	N	6.18
1	H	285:VAL	C	302:SER	N	6.01
1	C	207:ALA	C	209:ALA	N	3.35

PRELIMINARY VALIDATION REPORT

6 Fit of model and data (i)

6.1 Protein, DNA and RNA chains (i)

In the following table, the column labelled '#RSRZ> 2' contains the number (and percentage) of RSRZ outliers, followed by percent RSRZ outliers for the chain as percentile scores relative to all X-ray entries and entries of similar resolution. The OWAB column contains the minimum, median, 95th percentile and maximum values of the occupancy-weighted average B-factor per residue. The column labelled 'Q< 0.9' lists the number of (and percentage) of residues with an average occupancy less than 0.9.

Mol	Chain	Analysed	<RSRZ>	#RSRZ>2			OWAB(Å^2)	Q<0.9
1	B	204/204 (100%)	0.52	1 (0%)	90	91	40, 62, 102, 127	0
2	A	202/202 (100%)	0.39	2 (0%)	82	84	39, 57, 96, 126	0
3	D	202/202 (100%)	0.54	7 (3%)	44	48	36, 66, 107, 163	0
4	C	188/188 (100%)	1.04	28 (14%)	3	2	57, 98, 135, 164	0
5	E	201/201 (100%)	0.87	22 (10%)	6	6	66, 93, 120, 167	0
6	H	89/89 (100%)	3.98	64 (71%)	0	0	127, 155, 176, 184	0
All	All	1086/1086 (100%)	0.94	124 (11%)	6	5	36, 79, 153, 184	0

All (124) RSRZ outliers are listed below:

Mol	Chain	Res	Type	RSRZ
6	H	260	LEU	15.6
6	H	125	LEU	13.3
6	H	194	VAL	9.6
6	H	262	MET	9.4
6	H	303	ILE	9.2
6	H	284	LEU	8.7
6	H	265	ALA	8.6
6	H	124	CYS	8.0
6	H	275	PRO	7.9
6	H	219	LEU	7.7
6	H	285	VAL	7.3
6	H	193	MET	7.2
5	E	224	SER	7.0
6	H	126	LEU	7.0
4	C	206	LEU	6.4
6	H	269	CYS	6.3
6	H	142	ILE	5.8
5	E	240	ARG	5.7
6	H	192	GLN	5.7

Continued on next page...

PRELIMINARY VALIDATION REPORT

Continued from previous page...

Mol	Chain	Res	Type	RSRZ
6	H	326	ALA	5.7
6	H	325	GLU	5.6
6	H	306	ILE	5.5
6	H	146	ILE	5.3
6	H	141	LEU	5.2
6	H	302	SER	5.2
3	D	208	GLU	5.0
6	H	310	ILE	5.0
4	C	248	ILE	4.9
6	H	264	MET	4.9
6	H	157	LEU	4.8
4	C	240	ARG	4.8
6	H	135	LYS	4.7
6	H	218	VAL	4.7
6	H	267	GLU	4.6
6	H	144	ARG	4.6
6	H	137	VAL	4.6
6	H	134	GLY	4.5
6	H	266	THR	4.5
6	H	327	LEU	4.5
6	H	280	ARG	4.5
6	H	196	THR	4.4
6	H	138	ALA	4.4
4	C	299	VAL	4.2
4	C	210	GLY	4.1
4	C	205	SER	4.1
6	H	263	ALA	4.1
6	H	148	GLU	4.1
6	H	123	VAL	4.1
6	H	261	ASN	4.1
6	H	268	MET	4.1
5	E	126	LEU	4.0
3	D	207	ALA	4.0
6	H	283	PRO	4.0
6	H	122	PRO	4.0
6	H	213	PHE	3.8
3	D	205	SER	3.6
5	E	142	ILE	3.6
4	C	126	LEU	3.5
6	H	143	ALA	3.5
4	C	163	HIS	3.5
5	E	237	ALA	3.4

Continued on next page...

PRELIMINARY VALIDATION REPORT

Continued from previous page...

Mol	Chain	Res	Type	RSRZ
6	H	311	ILE	3.4
4	C	209	ALA	3.4
6	H	274	GLN	3.4
6	H	145	ALA	3.4
6	H	120	ILE	3.3
3	D	325	GLU	3.3
4	C	247	ASP	3.3
5	E	238	LEU	3.2
4	C	245	ASP	3.2
4	C	193	MET	3.2
6	H	139	THR	3.1
5	E	262	MET	3.1
6	H	307	THR	3.0
5	E	280	ARG	2.9
4	C	306	ILE	2.9
5	E	178	LEU	2.9
6	H	220	ALA	2.9
5	E	268	MET	2.8
5	E	260	LEU	2.8
4	C	211	ILE	2.8
6	H	215	SER	2.8
6	H	282	CYS	2.8
6	H	320	ILE	2.8
6	H	214	THR	2.7
6	H	315	ASN	2.6
4	C	204	ALA	2.6
6	H	276	ALA	2.6
6	H	136	SER	2.6
6	H	195	SER	2.6
5	E	187	MET	2.6
5	E	145	ALA	2.5
6	H	323	CYS	2.5
5	E	154	THR	2.5
4	C	178	LEU	2.5
4	C	242	PHE	2.5
5	E	257	ASP	2.5
5	E	125	LEU	2.4
4	C	199	PHE	2.4
4	C	176	ASP	2.4
5	E	329	GLN	2.4
6	H	147	ALA	2.4
4	C	324	MET	2.4

Continued on next page...

PRELIMINARY VALIDATION REPORT

Continued from previous page...

Mol	Chain	Res	Type	RSRZ
3	D	224	SER	2.4
4	C	190	PHE	2.4
5	E	250	VAL	2.3
4	C	146	ILE	2.3
4	C	135	LYS	2.2
4	C	175	MET	2.2
4	C	200	ILE	2.2
5	E	310	ILE	2.2
5	E	173	VAL	2.2
5	E	314	ARG	2.2
4	C	300	ARG	2.1
2	A	235	SER	2.1
6	H	149	ALA	2.1
6	H	321	GLY	2.1
1	B	225	SER	2.1
4	C	161	PRO	2.1
5	E	247	ASP	2.0
3	D	248	ILE	2.0
2	A	238	LEU	2.0
4	C	311	ILE	2.0
3	D	161	PRO	2.0

6.2 Non-standard residues in protein, DNA, RNA chains (i)

There are no non-standard protein/DNA/RNA residues in this entry.

6.3 Carbohydrates (i)

There are no carbohydrates in this entry.

6.4 Ligands (i)

In the following table, the Atoms column lists the number of modelled atoms in the group and the number defined in the chemical component dictionary. LLDF column lists the quality of electron density of the group with respect to its neighbouring residues in protein, DNA or RNA chains. The B-factors column lists the minimum, median, 95th percentile and maximum values of B factors of atoms in the group. The column labelled 'Q< 0.9' lists the number of atoms with occupancy less than 0.9.

Mol	Type	Chain	Res	Atoms	RSCC	RSR	LLDF	B-factors(Å^2)	Q<0.9
7	ZN	F	1	1/?	1.00	0.21	1.44	62,62,62,62	0

Continued on next page...

PRELIMINARY VALIDATION REPORT

Continued from previous page...

Mol	Type	Chain	Res	Atoms	RSCC	RSR	LLDF	B-factors(Å^2)	Q<0.9
7	ZN	F	5	1/?	0.98	0.18	0.61	94,94,94,94	0
7	ZN	F	3	1/?	0.99	0.18	0.46	72,72,72,72	0
8	PO4	G	3	5/?	0.80	0.20	0.36	68,90,100,110	0
7	ZN	F	7	1/?	0.98	0.20	0.35	56,56,56,56	0
7	ZN	F	4	1/?	0.99	0.17	-0.06	65,65,65,65	0
8	PO4	G	2	5/?	0.95	0.17	-0.13	71,74,77,84	0
8	PO4	G	1	5/?	0.94	0.13	-1.82	78,87,96,102	0
7	ZN	F	6	1/?	0.32	0.10	-	233,233,233,233	0

6.5 Other polymers (i)

There are no such residues in this entry.

PRELIMINARY VALIDATION REPORT
